# MicroRNAs-Based Theranostics against Anesthetic-Induced Neurotoxicity

**DOI:** 10.3390/pharmaceutics15071833

**Published:** 2023-06-27

**Authors:** Roseleena Minz, Praveen Kumar Sharma, Arvind Negi, Kavindra Kumar Kesari

**Affiliations:** 1 Department of Life Sciences, Central University of Jharkhand, Brambe, Ranchi 853205, Jharkhand, India; 2 Department of Bioproducts and Biosystems, School of Chemical Engineering, Aalto University, 02150 Espoo, Finland; 3 Department of Applied Physics, School of Science, Aalto University, 02150 Espoo, Finland

**Keywords:** miRNA, neurotoxicity, antagomir, agomir, anesthetic neurotoxicity

## Abstract

Various clinical reports indicate prolonged exposure to general anesthetic-induced neurotoxicity (in vitro and in vivo). Behavior changes (memory and cognition) are compilations commonly cited with general anesthetics. The ability of miRNAs to modulate gene expression, thereby selectively altering cellular functions, remains one of the emerging techniques in the recent decade. Importantly, engineered miRNAs (which are of the two categories, i.e., agomir and antagomir) to an extent found to mitigate neurotoxicity. Utilizing pre-designed synthetic miRNA oligos would be an ideal analeptic approach for intervention based on indicative parameters. This review demonstrates engineered miRNA’s potential as prophylactics and/or therapeutics minimizing the general anesthetics-induced neurotoxicity. Furthermore, we share our thoughts regarding the current challenges and feasibility of using miRNAs as therapeutic agents to counteract the adverse neurological effects. Moreover, we discuss the scientific status and updates on the novel neuro-miRNAs related to therapy against neurotoxicity induced by amyloid beta (Aβ) and Parkinson’s disease (PD).

## 1. Introduction

MicroRNAs (miRNAs or μRNAs) are conserved, small, endogenous, non-coding RNAs of approximately 21 to 23 nucleotides [1] and highly conserved across higher eukaryotes. The miRNAs are synthesized in the nucleus, as pri-miRNAs with the help of RNA polymerase II, and then processed by a complex of endoribonuclease and RNA-binding partner or by components of the splicing machinery [2]. The pre-miRNAs are exported to the cytoplasm and are further processed by endoribonuclease DICER and RNA-binding proteins, TRBP and PACT. This processing results in double-stranded miRNA duplexes which are loaded into the RNA-induced silencing complex (RISC). The miRNA interacts with its target mRNA in a process mediated by argonaute-2 (AGO2) and chaperones and carries out either post-translational gene regulation or target mRNA degradation, thus leading to gene silencing [3,4]. Furthermore, miRNAs can be exported and imported by cells using extracellular vesicles (EVs) or as a part of the protein–miRNA complex, and during this process, miRNAs may also be detected in bodily fluids [5]. In addition to export, some miRNAs in bodily fluids may originate from broken or damaged cells and are stable to be detected in the blood, urine, or other body fluids.

Some of them are identified as key gene regulators; those (miRNAs) can be exploited as therapeutic and diagnostic tools. Targeting miRNA-mediated gene networks in different components of the tumor microenvironment (cancer cells and the surrounding cellular and non-cellular components that interact with each other) holds promise for novel cancer treatments and improved therapeutic responses [6]. For example, an increased abundance of let-7 miRNA has been associated with a positive response to anti-epidermal growth factor receptor (EGFR) therapy in colorectal cancer (CRC) patients. Conversely, miRNA-21 has been implicated in promoting resistance to 5-fluorouracil (FU) chemotherapy, and inhibitors of this miRNA are being evaluated for the treatment of CRC and other cancers [7]. A survey of databases performed on 19th June 2020 by one of the authors has retrieved 7055 US patents, 5280 European patents, and 87,700 Google patents linked with miRNA therapeutic applications. Those patents were associated with the application of miRNA in cancer. Amid synthetic miRNA oligos (oligonucleotides), Miravirsen (SPC3649) targeting miR-122 for hepatitis C virus (HCV) treatment has entered phase II clinical trials under the biopharmaceutical company SantarisPharma, Copenhagen, Denmark. MRX34 (for cancer treatment targeting miR-34), Cobomarsen (MRG-106) (for cutaneous T-cell lymphoma treatment targeting miR-155), MRG-107 (for amyotrophic lateral sclerosis treatment targeting miR-155), MRG-110 (for ischemia treatment targeting miR-92a), and Remlarsen (MRG-201) (for fibrosis treatment targeting miR-29) are under development by miRagen therapeutics, Colorado, US, while RG-101 (for viral effect targeting miR-122) and RGLS4326 (polycystic kidney disease treatment targeting miR-17) by Regulus Therapeutics, California, USA, are in the stage of miRNA therapeutics phase 1 clinical trial. Furthermore, the development of various miRNA delivery systems, such as polymeric vectors, atelocollagen (ATE), poly lactic-co-glycolic acid (PLGA), polyamidoamine (PAMAM), degradable dendrimers, inorganic nano-materials, lipid-based delivery systems, viral vectors, and advance red blood cell extracellular vesicles (O-RBCs) has improved the preciseness of synthetic miRNA oligos towards its target [8]. Currently, there is ongoing biopharmaceutical research focused on enhancing the pharmacokinetics (ADMET: absorption, distribution, metabolism, excretion, and toxicity) of miRNA using various delivery systems, demonstrating the growing interest of multinational pharmaceutical companies in developing miRNA-based treatments. 

## 2. miRNAs and Neurotoxicity

Environmental factors associated with neurotoxicity (including day-to-day life events) are often deceiving to people and detected (in some instances) only in prolonged exposure or in advanced stages, therefore require efficient diagnosis methods [9]. Moreover, the detection of neurotoxicity needs repetitive studies (via suitable clinical models), high-throughput screening, and a search for relevant therapeutic criteria. Conventionally, neurotoxicity can be detected by observing the changes in individual behavior (or physical activity), electrophysiology, and histopathological processing of brain tissues [10,11,12,13,14,15,16,17]. However, these traditional neurotoxicity assessments are often associated with invasive sampling or lack of sensitivity, specificity, quantitative matrix, preclinical detection, targeted therapeutic approaches, and lack of understanding of etiology connections (or mechanisms) [18]. The miRNAs present in the brain tissues and CSF (cerebrospinal fluid) act as the critical regulator of neuronal gene expression implicated in brain development, neuronal and glial cell functions [19], cognition, synaptic plasticity, and spatial and temporal properties of neurons [20]. miRNA-based neurotoxicity assessment having specificity, sensitivity, and quantitative approach along with novel modification not only represents an ideal approach towards the challenging assessment of silent neurotoxicity but also opens up new avenues of therapeutic intervention in neurotoxicity.

To evaluate the status of the potential miRNAs associated with neurotoxicity, we went through a literature search (using the PubMed database (https://pubmed.ncbi.nlm.nih.gov/ (accessed on 25 September 2022))) by using the keyword “neurotoxicity AND miRNAs.” We used the literature published in 5 years, from 2017 to 2022 (till 25 September 2022), to acknowledge the recent updates and trends in this field. This search led to the retrieval of 328 papers. These papers were then screened based on their relevance and suitability to the research question, and documents that did not focus on the association between miRNAs and neurotoxicity were excluded. After the screening process, out of the identified 72 published studies that investigated remedial approaches related to neurotoxicity, 30 published studies that rely on potential miRNAs as alleviative targets for anesthetic neurotoxicity were explored to understand the engineered miRNA-based possible strategies and their implications in anesthetic neurotoxicity. The details of screened studies for potential miRNAs as alleviative targets for neurotoxicity and miRNA modulators towards neurotoxicity are summarized in Supplementary Tables S1 and S2, respectively. We performed the literature search on PubMed (https://pubmed.ncbi.nlm.nih.gov/ (accessed on 25 September 2022)) by using the keyword “neurotoxicity AND miRNAs,” which resulted in 329 papers for 5 years (2017–2022) of duration. Out of these publications, 72 published studies rely on a remedial approach related to neurotoxicity and 46 published studies targeted the different miRNA modulators towards neurotoxicity (Table 1 and Table 2).

The report of neurotoxicity induced by anesthetics and heavy metals included in the study was based on animal models and cell lines. Contrary to this, evidence of neurotoxicity related to Alzheimer’s disease (AD) and Parkinson’s disease (PD) originated from studies in animal models, cell lines as well as plasma, serum, peripheral blood, and cerebrospinal fluid (CSF) [21,22,23,24,25,26,27,28]. 

**Figure 1:**
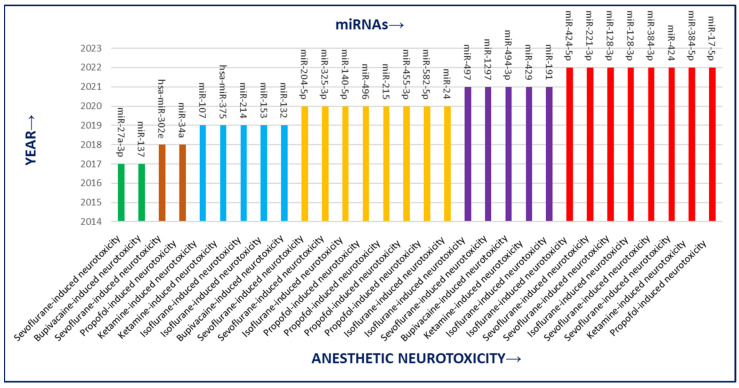
Study status (2017–2022) for anesthesia-induced neurotoxicity under potential approach for neurotoxicity alleviation via miRNA.

**Figure 2:**
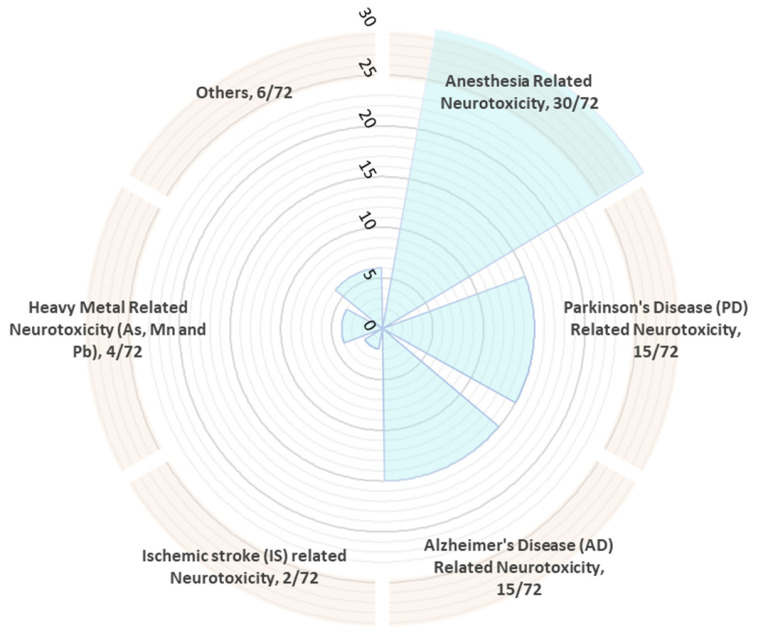
Literature study frequency for the miRNA-based alleviative target for neurotoxicity of 5 years (2017–2022).

Out of 72 published literature studies analyzed, the study frequency score for anesthetic-related neurotoxicity was highest, i.e., 30/72, while for ischemic stroke (IS)-related neurotoxicity was lowest, i.e., 2/72. Furthermore, AD, PD, heavy metal-induced, and other forms of neurotoxicity frequency were found to be 15/72, 15/72, 4/72, and 6/72, respectively (Figure 1 and Figure 2). Neurotoxicity induced by anesthetics included sevoflurane-induced, bupivacaine-induced, ketamine-induced, propofol-induced, and isoflurane-induced neurotoxicity. The AD patients suffer from neurotoxicity due to amyloid-β peptide, and PD patients have 6-hydroxydopamine, 1-methyl-4-phenylpyridinium/MPP(+)-induced and atrazine-induced neurotoxicity. Heavy-metals-induced neurotoxicity is related to arsenic (As) and lead (Pb). Other neurotoxicants included glutamate-induced neurotoxicity, triazophos-induced toxicity, METH-mediated neurotoxicity, T helper cell 1 (Th1)-skewed neurotoxicity, lidocaine-induced neurotoxicity, and oxygen-glucose deprivation/reoxygenation (OGD/R)-induced neurotoxicity.

There is corroborating evidence linking the involvement of miRNAs in the regulation of neuronal apoptosis and neurogenesis and they might be a crucial therapeutic–diagnostic factor to direct “neurotoxicity attenuation” via specific targets and pathways. As per our literature search, 29 miRNAs have their role in anesthetic neurotoxicity (Table 1), 16 miRNAs are associated with AD-related neurotoxicity,14miRNAs had been linked with PD-related neurotoxicity, and 2 miRNAs are associated with ischemic stroke (IS)-related neurotoxicity (Supplementary Tables S3–S5). Other types of miRNAs and their association had been listed in Supplementary Tables S6 and S7.

**Table 1:** Potential miRNAs as the alleviative target for anesthetic neurotoxicity.

Sr. No.	Anesthetic Neurotoxicity	miRNA	Targets/Signaling Pathways	Experimental Validation Approach	References
1.	Sevoflurane-induced neurotoxicity	miR-27a-3p	PPAR-γ signaling pathway	Mouse model	[29]
2.	Bupivacaine-induced neurotoxicity	miR-137	LSD1	Cultured in vitro Murine DRGNs	[30]
3.	Sevoflurane-induced neurotoxicity	hsa-miR-302e	OXR1	Human hippocampal cells (HN-h)	[31]
4.	Propofol-induced neurotoxicity	miR-34a	MAPK/ERK signaling pathway	In vivo and in vitro (Sprague–Dawley rats and SH-SY5Y cells)	[32]
5.	Ketamine-induced neurotoxicity	miR-107	BDNF	ESC-derived neurons	[33]
6.	Ketamine-induced neurotoxicity	hsa-miR-375	BDNF	Human embryonic stem cell (hESC)-derived neuron model	[34]
7.	Isoflurane-induced neurotoxicity	miR-214	PTEN	Human neuroblastoma cell line SH-SY5Y	[35]
8.	Isoflurane-induced neurotoxicity	miR-153	Nrf2/ARE	Vitro mice model	[36]
9.	Bupivacaine-induced neurotoxicity	miR-132	IGF1R	Human neuroblastoma cell line (SH-SY5Y)	[37]
10.	Sevoflurane-induced neurotoxicity	miR-204-5p	BDNF/TrkB/Akt pathway	Mouse hippocampal neuronal cell line (HT22)	[38]
11.	Sevoflurane-induced neurotoxicity	miR-325-3p	Nupr1 and C/EBPβ/IGFBP5 signaling	Neonatal rats and HCN-2 human cortical neuronal cells	[39]
12.	Isoflurane-induced neurotoxicity	miR-140-5p	SNX12	Diabetic rat model	[40]
13.	Propofol-induced neurotoxicity	miR-496	ROCK2	Primary prefrontal cortical (PFC) neurons of neonatal rats	[41]
14.	Propofol-induced neurotoxicity	miR-215	LATS2	Neonatal rat hippocampal neuron	[42]
15.	Propofol-induced neurotoxicity	miR-455-3p	EphA4	Primary hippocampal neurons of SD (Sprague–Dawley) rats	[43]
16.	Propofol-induced neurotoxicity	miR-582-5p	ROCK1	Primary rat hippocampal neurons	[44]
17.	Isoflurane-induced neurotoxicity	miR-24	p27kip1	Rat hippocampal neurons	[45]
18.	Isoflurane-induced neurotoxicity	miR-497	PLD1	Neonatal rat’s hippocampus and hippocampal primary neuronal cell	[46]
19.	Sevoflurane-induced neurotoxicity	miR-1297	PTEN	Mice	[47]
20.	Bupivacaine-induced neurotoxicity	miR-494-3p	CDK6-PI3K/AKT Signaling	Primary mouse hippocampal neuronal cells (C57BL/6 mice)	[48]
21.	Ketamine-induced neurotoxicity	miR-429	BAG5	PC12 cells	[49]
22.	Isoflurane-induced neurotoxicity	miR-191	BDNF	In vitro and in vivo (hippocampal tissues of rats)	[50]
23.	Isoflurane-induced neurotoxicity	miR-424-5p	FASN	hESC-derived neurons	[51]
24.	Sevoflurane-induced neurotoxicity	miR-221-3p	CDKN1B	Rat hippocampal neuron cells	[52]
25.	Sevoflurane-induced neurotoxicity	miR-128-3p	NOVA1	Rat hippocampal neuron cells	[53]
26.	Isoflurane-induced neurotoxicity	miR-128-3p	specificity protein 1 (SP1)	Sprague–Dawley (SD) rats	[54]
27.	Sevoflurane-induced neurotoxicity	miR-384-3p	Aak1	Rat hippocampus	[55]
28.	Sevoflurane-induced neurotoxicity	miR-424	TLR4/MyD88/NF-κB pathway	Mouse and in PC12 cells	[56]
29.	Ketamine-induced neurotoxicity	miR-384-5p	GABRB1	Neonatal hippocampal neurons from rats	[57]
30.	Propofol-induced neurotoxicity	miR-17-5p	BCL2L11	SH-SY5Y cells	[58]

## 3. Preclinical and Clinical Evidence on Anesthetic Neurotoxicity

FDA-approved halogenated inhalational sevoflurane is used to induce and maintain general anesthesia in adults and children undergoing *inpatient* and *outpatient* surgeries [59]. From the amide category of local anesthetics, bupivacaine is a strong local anesthetic for regional, epidural, spinal, and local infiltration anesthesia [60]. For quick medical procedures that do not need skeletal muscle relaxation, apply Ketamine as a pre-anesthetic medication alone or in conjunction with other drugs [61]. Similarly, propofol (an intravenous anesthetic) and isoflurane (FDA-approved volatile anesthetic) are used for general anesthesia induction, monitored anesthesia management, or procedural sedation.

Nonetheless, after the safety announcement released by the U.S. Food and Drug Administration (FDA) in 2016 (source: https://www.fda.gov/drugs/drug-safety-and-availability/2016-drug-safety-communications (accessed on 25 September 2022)), which stated that children who experience prolonged periods of anesthesia lasting over 3 h or receive multiple anesthesia treatments are at a heightened risk of developing future issues related to memory, learning, and behavior [62], the preclinical, experimental evidence is increasing. While clinical evidence from randomized controlled trials (RCTs) is limited due to ethical considerations, real-world reports and retrospective studies have examined anesthetics’ potential neurotoxicity (Table 2). Furthermore, study reports also link the risk of inhaled anesthetic neurotoxicity among the operating room personnel, patients, and anesthesiologists [63,64]. For instance, a recent study investigated the levels of toxic anesthetic gas isoflurane in the operating rooms of Valiasr and Shahid Beheshti teaching hospital during 2018 and assessed the associated health risks. The findings indicated that isoflurane levels exceeded the acceptable standard based on National Institute for Occupational Safety and Health (NIOSH) due to issues with the ventilation system [65]. These studies provide valuable insights. However, it is important to interpret these real-world reports and retrospective studies cautiously, as they may have limitations, such as selection bias, confounding factors, and inability to establish causation, and the evidence is still evolving. Continued research and investigation are necessary to refine our understanding of the risks and develop strategies to minimize potential adverse effects.

**Table 2:** Clinical evidence on anesthetic-based neurotoxicity: real-world reports and retrospective studies.

Real-World Reports and Retrospective Studies		Study Type	References
Mayo Clinic Study (Rochester, MN, USA)—1976 to 1982	A retrospective study at the Mayo Clinic examined the medical records of children who had undergone multiple surgeries with anesthesia before age 4. The study found a correlation between repeated exposure to anesthesia and a higher risk of developing learning disabilities (LD) and developmental disorders later in childhood. In contrast, the data from the study do not provide evidence as to whether anesthesia contributes to the development of LD or if the need for anesthesia serves as an indicator for other unknown factors associated with LD.	Population-based birth cohort study	[66]
Taiwan National Health Insurance Research Database (NHIRD) Study—2001 to 2005	This population-based study analyzed data from the Taiwan National Health Insurance Research Database under large longitudinal observation and sample size and included over 3293 out of 16,465 children who underwent surgery before the age of 3. The study found that exposure to GA before the age of 3 was not associated with an increased risk of ADHD.	Population-based/ matched cohort study	[67]
GAS Trial Study—2007 to 2013: Neurodevelopmental outcome at age 2	This GAS trial aimed to determine whether general anesthesia in infancy affects neurodevelopmental outcomes. Infants undergoing inguinal herniorrhaphy were randomly assigned to receive either awake-regional anesthesia or general anesthesia with sevoflurane. The primary outcome, assessed at age 5, is the WPPSI-III Full Scale Intelligence Quotient score. The secondary outcome, reported here, assessed cognitive development at 2 years using the composite cognitive score from the Bayley Scales of Infant and Toddler Development III. The analysis revealed no significant difference in cognitive scores between the two anesthesia groups, suggesting that administering sevoflurane anesthesia for less than 1 h during infancy does not increase the risk of adverse neurodevelopmental outcomes compared to awake-regional anesthesia.	General Anesthesia compared to Spinal anesthesia (GAS) trial	[68]
Pediatric Anesthesia and Neurodevelopment Assessment (PANDA) Study—2009 to 2015	This sibling-matched cohort study aimed to examine the potential long-term effects of a single anesthesia exposure in otherwise healthy young children involving 105 pairs of siblings aged 8 to 15 years. The exposed siblings had undergone a single anesthesia exposure during inguinal hernia surgery before the age of 36 months, while the unexposed siblings had no history of anesthesia exposure. The neurocognitive and behavior outcomes were assessed prospectively, with anesthesia exposure data documented retrospectively. There were no significant differences in domain-specific neurocognitive functions (such as memory/learning, motor/processing speed, visuospatial function, attention, executive function, and language) or behavior between the exposed and unexposed sibling pairs. Based on these findings, the study concluded that a single anesthesia exposure before the age of 36 months in healthy children did not result in significant differences in IQ scores or neurocognitive function in later childhood. However, the researchers emphasized the need for further investigation into the effects of repeated or prolonged anesthesia exposure, as well as the potential vulnerability of certain subgroups of children.	Sibling-matched cohort study/PANDA trial	[69]
Western Australian Pregnancy Cohort (Raine) Study—1989 to 1992	This prospective cohort study on clinical phenotype followed over 1444 children from birth to the age of 10. The study investigated the association between early exposure to anesthesia and surgery and long-term neurodevelopmental deficits in children. The cohort was divided into four subclasses based on neurodevelopmental deficits: Normal, Language and Cognitive deficits, Behavioral deficits, and Severe deficits. The results showed that children in the Language and Cognitive deficit group were more likely to have been exposed to anesthesia and surgery before the age of 3. However, there was no significant difference in exposure between the Behavioral or Severe deficit groups and the Normal group. The findings suggest that the phenotype of interest in evaluating children exposed to anesthesia and surgery should focus on deficits primarily in language and cognition, rather than broad neurodevelopmental delay or primarily behavioral deficits.	Population-based cohort study	[70]
Mayo Clinic Study (Rochester, MN, USA)—1996 to 2000	This new birth cohort study via modern techniques investigated whether undergoing multiple procedures requiring general anesthesia (GA) before the age of 3 is linked to negative neurodevelopmental outcomes. They analyzed data from 116 children with multiple exposures, 457 with single exposures, and 463 with no exposures. The results showed that multiple exposures were associated with a higher frequency of both LD and attention-deficit hyperactivity disorder (ADHD), compared to the unexposed group with a hazard ratio (HR) for LD of 2.17. Multiple exposures were also associated with lower cognitive ability and academic achievement. On the other hand, single exposures were only modestly linked to decreases in reading and language achievement, without affecting cognitive ability significantly. These findings, which align with a previous study on an older cohort, provide further evidence that children with multiple exposures to anesthesia are more likely to experience adverse outcomes in terms of learning and attention.	Population-based birth cohort study	[71]
Mayo Anesthesia Safety in Kids (MASK) Study—1994 to 2007	This retrospective cohort study analyzed data from over 411 unexposed, 380 singly exposed, and 206 multiply exposed children with anesthesia before the age of 3. The study concluded that exposure to anesthesia was not associated with deficits in general intelligence. However, multiple exposures were linked to slight reductions in processing speed and fine motor coordination, as well as increased difficulties in behavior and reading according to parent reports. These secondary outcomes should be interpreted cautiously, but they suggest a hypothesis that multiple anesthesia exposures may cause specific changes in certain neuropsychological domains, potentially leading to behavioral and learning difficulties. Further research is needed to validate these findings and explore the long-term implications.	Population-based study	[72]
GAS Trial Study—2007 to 2013: Neurodevelopmental outcome at age 5	This international GAS trial was a multicenter RCT conducted to compare the neurodevelopmental outcomes of infants undergoing hernia repair under general anesthesia versus regional anesthesia. A follow-up study assessed the neurodevelopmental outcomes of the children at the age of 5. The findings concluded that the administration of slightly less than 1 h of general anesthesia in early infancy did not have a significant impact on neurodevelopmental outcomes at 5 years of age compared to awake-regional anesthesia. These results were consistent across the predominantly male study population.	GAS trial	[73]
General Anesthesia and Cognitive Decline (GACD) Study—2004 to 2009	This study was conducted on 1819 older adults to analyze their cognitive function over time. The study compared the rate of cognitive decline in participants exposed to regional anesthesia (RA) or general anesthesia (GA) with those who were not exposed to any anesthesia. The results showed that compared to those unexposed to anesthesia, both RA and GA were associated with a greater rate of decline in overall cognitive function over time. The rates of decline were similar for both RA and GA and did not differ significantly. However, when looking at specific cognitive domains, a faster decline in memory was observed in participants who received GA but not in those who received RA. The observed decline in memory associated with GA needs further confirmation before any conclusions about mechanisms or changes in practice can be made.	Population-based study	[74]
Taiwan NHIRD Study—2000 to 2013	In a compared group of 11,457 children who received general anesthesia before the age of 2 to a group of 22,914 children who were not exposed to anesthesia, this study revealed that longer total anesthesia durations were associated with an elevated risk of developmental delay (DD). Among children with anesthesia durations of less than 2 h, the HR was 1.124, indicating a 12.4% increased risk. For anesthesia durations between2 and 4 h, the HR was 1.450, representing a 45% increased risk. Moreover, for anesthesia durations exceeding 4 h, the HR was 1.598, indicating a 59.8% increased risk.	National population-based cohort study	[75]

## 4. Engineered miRNA to Attenuate Anesthetic Neurotoxicity

The emergence of “engineered miRNAs," a pre-designed synthetic miRNA sequence, might be a “reverting substitute” against highly specific miRNAs. Engineered miRNAs in the form of “agomir” (ds oligos/double-strand oligonucleotides) have the efficiency to mimic the role of suppressed miRNA. In contrast, “antagomir” (ss oligos/single-strand oligonucleotides) directs the suppression of overexpressed miRNA. Additionally, as a "mini-regulating element," it can efficiently regulate the level of apoptotic factors, cytokines, and oxidative stress enzymes in addition to specific signaling pathways and gene expression. It centers the “retrograde motion” to understand, regulate, or modulate the miRNA-based mechanisms. The ss oligos-antagomirs are saline-soluble and can be intravenous (IV) and subcutaneous (SC) administrative drugs. However, unlike the synthetic siRNA oligo, the challenging factor for miRNA oligo is “TMTME” (too many targets for the miRNA effect) [76]. Contrary to this, delivering ds oligos-agomir in nanocarrier (such as exosomes, vectors, RNA sponges, and lentivirus) can be more effective in reaching the specific target.

Mechanisms such as neuroapoptosis, splicing, oxidative stress, and neuroplasticity have been implicated in miRNA-dependent neurotoxicity. These mechanisms involve specific target genes, signaling pathways, and signaling cascades. For example, miRNA-dependent APP (amyloid precursor protein) neurotoxicity is a splicing-dependent process in AD pathology and involves miR-101, miR-20a, miR-17-5p, miR-106b, miR-106a, miR-520c, miR-16, miR-124, miR-147, miR-153, miR-644, and miR-323. Furthermore, miR-107, miR-29a, miR-29b-1, miR-9, miR-15, miR-29c, miR-298, miR-328, miR-195, and miR-124 regulate the expression of BACE 1(β-site APP-cleaving enzyme), an enzyme [77,78] involved in Aβ plaques aggregation. The α-synuclein aggregation that mediates toxicity in PD is dependent on chaperon-mediated autophagy (miR-214, miR-7, miR-34b/c, miR-153, miR-26b, miR-301b, miR-106a, miR-16-1, miR-320a, miR-21, miR-373, miR-379, and miR-224) [77]. In addition, the literature studies reveal that neuroapoptosis paves the common miRNA-mediated neurotoxicity mechanism for anesthetic-stimulant neurotoxicity. 

The inhibitory mechanism implicated through the “chemically engineered miRNA” known as “miRNA agomir/miRNA antagomirs” to suppress and revert the neurotoxicity pathway can be the promising therapeutic approach to neutralize the anesthetic neurotoxic effect. Several potential miRNAs against neurotoxicity are being experimentally analyzed to pave the miRNA-based attenuation mechanism. We have retrieved 30 engineered miRNAs (17 agomir/miRNA mimics and 13 antagomirs/miRNA inhibitors) against miRNA-based anesthetic neurotoxicity; a total of 9 engineered miRNAs against sevoflurane-induced neurotoxicity; 3 engineered miRNAs against bupivacaine-induced neurotoxicity; 4 engineered miRNAs against ketamine-induced neurotoxicity; 6 engineered miRNAs against propofol-induced neurotoxicity; and 8 engineered miRNAs against isoflurane-induced neurotoxicity from the specific 30 selected studies. 

**Figure 3:**
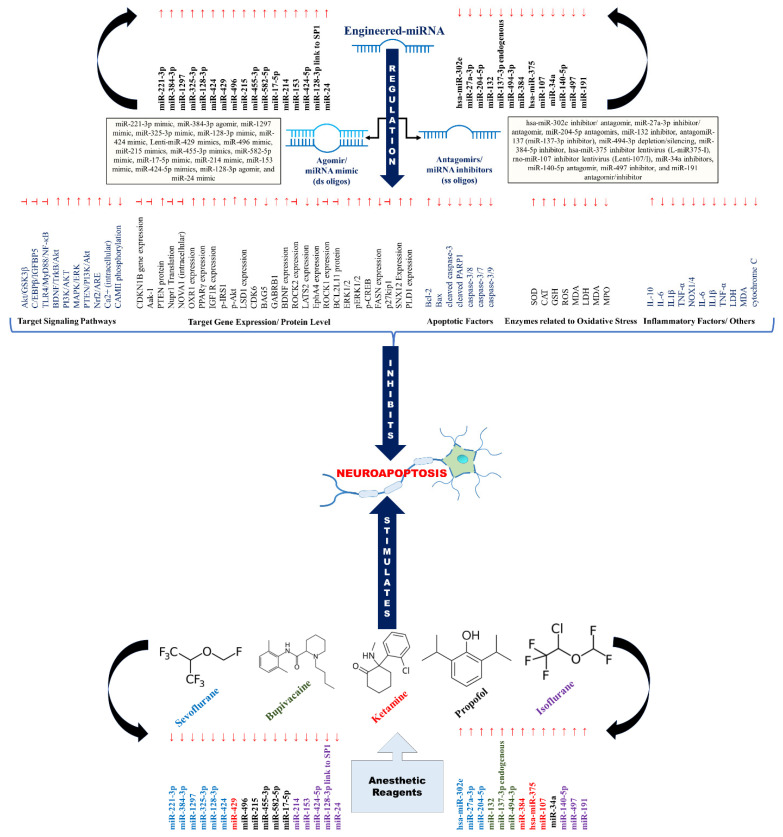
A schematic overview of attenuation mechanism against anesthesia-induced neurotoxicity via engineered miRNAs (agomir/antagomir) (**↑** = upregulation/activation/stimulation; ↓ = downregulation; **┴** = inhibition/inactivation ■ = sevoflurane ■ = bupivacaine ■ = ketamine ■ = propofol ■ = isoflurane).

These case studies reveal that the agomir/miRNA mimics can potentially enhance miRNA expression. In contrast, the antagomirs/miRNA inhibitors suppress the miRNA expression via regulation of specific target signaling pathways and target gene expression/protein level, as well as apoptotic factors, enzymes related to oxidative stress, inflammatory factors, and others. This directs the inhibition of neuroapoptosis stimulated by anesthetic agents (Figure 3).

For example, the agomir lenti-miR-429 mimic, miR-215 mimic, miR-214 mimic, miR-153 mimic, miR-424-5p mimic, and miR-24 mimic contribute to the upregulation of SOD, CAT, GSH, and downregulation of ROS, MDA, LDH, MDA, and MPO to suppress the oxidative stress. Then, the upregulation of anti-apoptotic factor-Bcl-2 and downregulation of pro-apoptotic factors (Bax, cleaved caspase-3, cleaved PARP1, caspase-3/8, caspase-3/7, and caspase-3/9) by agomirs (miR-221-3p mimic, miR-128-3p mimic, miR-424 mimic, lenti-miR-429 mimics, miR-214 mimic, miR-153 mimic, miR-424-5p mimics, and miR-24 mimic) and antagomirs (miR-204-5p antagomirs, miR-132 inhibitor, miR-34a inhibitors, miR-140-5p antagomir, and miR-497 inhibitor) signify the positive predictive marker towards neuroapoptosis suppression. Similarly, the regulation of inflammatory factors by agomir (miR-128-3p mimic, miR-424 mimic, and miR-24 mimic) and antagomir-hsa-miR-302e includes the upregulation of IL-10 and the downregulation of IL-6, IL1β, TNF-α, NOX1/4, IL-6, IL1β, TNF-α, LDH, MDA, and cytochrome c. The specific signaling pathways and targets to execute the inhibition of neuroapoptosis have been mentioned in Table 3.

## 5. Conclusions

Various challenges to achieving clinical success of miRNA-based theranostics are flawed with shortcomings, such as minimization of TMTME biases, cell-specific delivery and uptakes, production of synthetic miRNA substitutes, and its diagnostic and prognostic efficiency [79,80]. Nevertheless, the emergence of high-throughput screening and the recent advancement in synthetic medicinal chemistry strategies (efficient stereochemical synthetic routes, conjugate chemistry, and macromolecular designing) [81,82,83], to develop miRNA therapeutic molecules (notably, mini-oligo-nucleotides RNA-PROTACs [84,85], small-molecule inhibitors, antisense oligonucleotides [86], miR-mask oligonucleotides, miRNA sponges, synthetic miRNAs, miRNAs based on viral constructs) improve their metabolic instability, therapeutic efficacy, target selectivity (mitigate on-target toxicity [87]), and cellular delivery [88]. For example, nanoencapsulation using polymeric interfaces enhances metabolic stability (seen to regulate the programming of blood–brain barrier permeability by hypoxia) [89,90]; application of dendrimers and similar precursor molecules (triphenyl pyridine cores) to improve in vivo and in vitro stability and cellular delivery (some potential applications can be evident with dendrimeric-miRNA nanoformulations against glioblastoma stem cells) [91,92,93,94]; meso/nano-sized dependent delivery of miRNA (using mesoporous silica nanoparticles to target tumors) [95,96,97,98]. However, to improve the detection and optical control over miRNA functioning, nanoribbon biosensors (detecting the miRNA in colorectal cancer) [99], light-activated circular morpholino oligonucleotides [100,101], electrochemical nanohybrid platforms (detecting the label-free miRNA) [102,103,104], and chemical surface modification of polymers-based formulation [105,106] were developed. 

This paper focused on demonstrating engineered miRNAs’ potential as a potential strategy to minimize anesthetic-induced neurotoxicity. Furthermore, reviewed literature (compiled in the paper) showed the clinical significance of engineered agomirs and antagomirs in animal models and cell lines (for conventional anesthetic drugs). However, further studies are still required to consolidate the clinical safety of such claims. 

Computational modeling and databases could help identify and validate miRNA targets [107]. However, the lack of an appropriate computational algorithm affects the reproducibility of such results; therefore, researchers continuously work to improve them and integrate the target prediction algorithms using experimental data [108,109,110]. Another challenge is achieving cell-specific delivery and uptake of miRNAs, which is essential for effective treatment [111]. 

Designing and producing synthetic miRNA substitutes also require molecular modeling approaches, where the incorporation of chemical substitutes (small-to-medium sized) to construct various molecular weighted oligonucleotides involves predicting secondary structures and target-binding specificity. Furthermore, with evolving bioinformatic tools, multi-omics data integration, and machine learning algorithms, our understanding of miRNA regulatory networks is improving, leading to accurate predictions of miRNA-target interactions.

## Figures and Tables

**Table 3:** Regulating components by engineered miRNAs (agomir and antagomir) against anesthetic neurotoxicity (sevoflurane-induced neurotoxicity, bupivacaine-induced neurotoxicity, ketamine-induced neurotoxicity, propofol-induced neurotoxicity, and isoflurane-induced neurotoxicity): (a) target signaling pathways; (b) target gene expression/protein level; (c) apoptotic factors; (d) enzymes related to oxidative stress; and (e) inflammatory factors/others.

Anesthetic Neurotoxicity	Engineered miRNA Type	miRNA Expression	Target Signaling Pathways	Target Gene Expression/Protein level	Apoptotic Factors	Enzymes Related to Oxidative Stress	Inflammatory Factors/Others
Sevoflurane-induced neurotoxicity	miR-221-3p mimic	miR-221-3p ↑	-	Inhibition CDKN1B gene expression	Bcl-2 ↑ Bax ↓ cleaved caspase-3 ↓	-	-
	miR-384-3p agomir	miR-384-3p ↑	-	Inhibition of Aak-1	-	-	-
	miR-1297 mimic	miR-1297 ↑	Inhibition of Akt/GSK3β signaling pathway	Activation of PTEN protein	-	-	-
	miR-325-3p mimic	miR-325-3p ↑	Inactivation of C/EBPβ/IGFBP5 Signaling pathways	Suppression of Nupr1 Translation	-	-	-
	miR-128-3p mimic	miR-128-3p ↑	-	Inhibition of intracellular NOVA1	Bcl-2 ↑ Bax ↓ cleaved caspase-3 ↓	-	IL-6 ↓ IL1β ↓ TNF-α ↓ NOX1/4 ↓
	miR-424 mimic	miR-424 ↑	Inhibition of TLR4/MyD88/NF-κB Signaling pathways	-	Bcl-2 ↑ Bax ↓ cleaved caspase-3 ↓	-	IL-10 ↑ IL-6 ↓ IL1β ↓ TNF-α ↓
	hsa-miR-302e inhibitor/antagomir	hsa-miR-302e ↓	intracellular Ca^2+^ ↓ CAMII phosphorylation ↓	Upregulation of OXR1 expression	-	-	LDH ↓ MDA ↓
	miR-27a-3p inhibitor/antagomir	miR-27a-3p ↓	-	Upregulation of PPARγ expression	-	-	-
	miR-204-5p antagomirs	miR-204-5p ↓	stimulation of BDNF/TrkB/Akt pathway	-	Bcl-2 ↑ Bax ↓ cleaved caspase-3 ↓	-	-
Bupivacaine-induced neurotoxicity	miR-132 inhibitor	miR-132 ↓	-	Upregulation of IGF1R expression, p-IRS1 and p-Akt	caspase 3 ↓ cleaved PARP1 ↓	-	-
	antagomiR-137 (miR-137-3p inhibitor)	endogenous miR-137-3p ↓	-	Upregulation of LSD1 expression	-	-	-
	miR-494-3p depletion/silencing	miR-494-3p ↓	Activation of PI3K/AKT pathway	Upregulation of CDK6	-	-	-
Ketamine-induced neurotoxicity	Lenti-miR-429 mimics	miR-429 ↑	-	Downregulation of BAG5	Bcl-2 ↑ Bax ↓ caspase-3 ↓	CAT ↑ SOD1 ↑	-
	miR-384-5p inhibitor	miR-384 ↓	-	Upregulation of GABRB1	-	-	-
	hsa-miR-375 inhibitor lentivirus (L-miR375-I)	hsa-miR-375 ↓	-	Upregulation of BDNF expression	-	-	-
	rno-miR-107 inhibitor lentivirus (Lenti-107/I)	miR-107 ↓	-	Upregulation of BDNF expression	-	-	-
Propofol-induced neurotoxicity	miR-496 mimic	miR-496 ↑	-	Inhibition of ROCK2 expression	-	-	-
	miR-215 mimics	miR-215 ↑	-	Downregulation of LATS2 expression	-	SOD ↑ ROS ↓ MDA ↓ LDH ↓	-
	miR-455-3p mimics	miR-455-3p ↑	-	Downregulation of EphA4 expression	-	-	-
	miR-582-5p mimic	miR-582-5p ↑	-	Inhibition of ROCK1 expression	-	-	-
	miR-17-5p mimic	miR-17-5p ↑	-	Suppression of BCL2L11 protein levels	-	-	-
	miR-34a inhibitors	miR-34a ↓	Activation of MAPK/ERK signaling pathway	Upregulation of ERK1/2, pERK1/2 and p-CREB ↑	Bax ↓ caspase-3/8 ↓	-	-
Isoflurane-induced neurotoxicity	miR-214 mimic	miR-214 ↑	Regulation of PTEN/PI3K/Akt pathway	-	caspase-3/7 ↓	SOD ↑ GSH ↑ MDA ↓	-
	miR-153 mimic	miR-153 ↑	Stimulation of Nrf2/ARE pathway	-	caspase-3/9 ↓	CAT ↑ SOD ↑ MDA ↓ MPO ↓	-
	miR-424-5p mimics	miR-424-5p ↑	-	Downregulation of FASN expression	Bcl-2 ↑ Bax ↓ caspase-3 ↓	SOD ↑ GSH ↑ MDA ↓	-
	miR-128-3p agomir	miR-128-3p ↑ link to SP1	-	-	-	-	-
	miR-24 mimic	miR-24 ↑	-	Inhibition of p27kip1	cleaved caspase-3 ↓ cleaved PARP ↓	CAT ↑ SOD ↑ GSH-Px ↑ MDA ↓	cytochrome C ↓
	miR-140-5p antagomir	miR-140-5p ↓	-	Upregulation of SNX12 Expression	Bcl-2 ↑ caspase-3 ↓	-	-
	miR-497 inhibitor	miR-497 ↓	-	Stimulate PLD1 expression	caspase-3 ↓	-	-
	miR-191 antagomir/inhibitor	miR-191 ↓	-	Upregulation of BDNF expression	-	-	-

## Data Availability

Not applicable.

## Supplementary Materials

The following supporting information can be downloaded at: https://www.mdpi.com/article/10.3390/pharmaceutics15071833/s1, Table S1: Potential miRNAs as alleviative target for neurotoxicity; Table S2: Potential modulator of miRNA as alleviative target for neurotoxicity; Table S3: Potential miRNAs as alleviative target for AD related neurotoxicity; Table S4: Potential miRNAs as alleviative target for PD related neurotoxicity; Table S5. Potential miRNAs as alleviative target for IS related neurotoxicity; Table S6: Potential miRNAs as alleviative target for heavy metals related neurotoxicity; Table S7: Potential miRNAs as alleviative target for other types of neurotoxicity.
