# Supplementary material for: MicroRNAs-Based Theranostics against Anesthetic-Induced Neurotoxicity
Source: Pharmaceutics. 2023 Jun 27;15(7):1833. doi: 10.3390/pharmaceutics15071833 (PMC10385075; doi:10.3390/pharmaceutics15071833)
Supplement: Supplementary file 1 [file pharmaceutics-15-01833-s001.zip › pharmaceutics-2432957-supplementary.pdf]

Supplementary Materials: MicroRNAs-Based Theranostics against Anesthetic-Induced Neurotoxicity

Roseleena Minz, Praveen Kumar Sharma, Arvind Negi and Kavindra Kumar Kesari

Table S1. Potential miRNAs as alleviative target for neurotoxicity.

Sr. No.	Neurotoxicity	miRNAs	Targets/ Signalling Pathways	Experimental Validation Approach	References
1.	Sevoflurane-induced neurotoxicity	miR-27a-3p	PPAR-γ signalling pathway	Mouse model	[1]
2.	MPP (+)-induced	miR-124-3p	STAT3	SH-SY5Y cells	[2]
3.	Bupivacaine-induced neurotoxicity	miR-137	LSD1	Cultured in vitro Murine DRGNs	[3]
4.	Glutamate-induced neurotoxicity	miR-219	CaMKIIγ	Rat hippocampal neurons	[4]
5.	β -amyloid-induced neurotoxicity	miR-16	BACE1	AD tissues and the cellular AD model of PC12	[5]
6.	Arsenic-induced - neurotoxicity	miR-219	CaMKII	Mouse hippocampus	[6]
7.	6-Hydroxydopamine-Induced Cell Model of PD	miR-124-3p	ANAX5	PC12 and SH-SY5Y cells	[7]
8.	Triazophos-induced toxicity	miR-217	nup43	Adult zebrafish and ZF4 cells	[8]
9.	Sevoflurane-induced neurotoxicity	hsa-miR-302e	OXR1	Human hippocampal cells (HN-h)	[9]
10	Parkinson's Disease	miR-124	MEKK3	BV2 cells	[10]
11.	Amyloid-β-induced neurotoxicity	miR-107	BDNF-TrkB signaling pathway	Mice	[11]
12.	Manganese induced neurotoxic	miR-7 and miR-433	SNCA and FGF-20 mRNA	SH-SY5Y cells	[12]
13.	Propofol-induced neurotoxicity	miR-34a	MAPK/ERK signaling pathway	In vivo and in vitro (Sprague-Dawley rats and SH-SY5Y cells)	[13]
14.	Ketamine-induced neurotoxicity	miR-107	BDNF	ESC-derived neurons	[14]
15.	MPTP-induced Parkinson's disease	miR-190	Nlrp3	PD mouse model and BV2 cells	[15]
16.	Ketamine-induced neurotoxicity	hsa-miR-375	BDNF	Human embryonic stem cell (hESC)-derived neuron model	[16]
17.	Aβ-induced neurotoxicity	miR-34a	Caspase-2	SH-SY5Y cells	[17]
18.	Aβ-induced neurotoxicity	miR-409-5p	Plek	APP/PS1 mice cortexes	[18]
19.	Atrazine-induced Parkinson's disease	miR-7	BDNF/α-syn axis	Peripheral blood and brain tissue in a rat model of PD	[19]
20.	METH-mediated neurotoxicity	miR-142a-3p/miR-155-5p	Peli1	BV2 cells and brain of mouse	[20]

21.	MPP (+)-induced neurotoxicity	miR-9-5p	SIRT1	SH-SY5Y cells	[21]
22.	MPP (+)- neurotoxicity	miR-200a and miR-204	Sirt1 and BCL2	PC12 cells	[22]
23.	Isoflurane-induced neurotoxicity	miR-214	PTEN	Human neuroblastoma cell line SH-SY5Y	[23]
24.	Parkinson's Disease	miR-326	KLK7-Mediated MAPK Signaling Pathway	Mice with PD	[24]
25.	Isoflurane-induced neurotoxicity	miRNA-153	Nrf2/ARE	Vitro mice model	[25]
26.	Bupivacaine-induced neurotoxicity	miR-132	IGF1R	Human neuroblastoma cell line (SH-SY5Y)	[26]
27.	Pb neurotoxicity	miR-106b-5p	XIAP	HT-22 and PC12 cells	[27]
28.	Aβ-induced neurotoxicity	miR-29c-3p	TNFAIP1/ NF-κB signalling pathway	Neuroblastoma Cells	[28]
29.	Parkinson's disease	miR-103a-3p	Parkin/Ambra1 signaling	SH-SY5Y cells	[29]
30.	T helper cell 1 (Th1)-skewed neurotoxicity	miR-494	HDAC2-STAT4 pathway	Blood from AIS (acute ischemic stroke) patients and controls	[30]
31.	Sevoflurane-induced neurotoxicity	miR-204-5p	BDNF/TrkB/Akt pathway	Mouse hippocampal neuronal cell line (HT22)	[31]
32.	Sevoflurane-induced neurotoxicity	miR-325-3p	Nupr1 and C/EBPβ/IGFBP5 signaling	Neonatal rats and HCN-2 human cortical neuronal cells	[32]
33.	Isoflurane-induced neurotoxicity	miR-140-5p	SNX12	Diabetic rat model	[33]
34.	Aβ (25-35)-induced neurotoxicity	miR-212	PDCD4/ PI3K/AKT signaling pathway	Plasma from AD patients, SH-SY5Y and IMR-32 cells	[34]
35.	Aß$_{25-35}$-induced	miR-33	Akt/mTOR pathway	SH-SY5Y cells	[35]
36.	Propofol-induced neurotoxicity	miR-496	ROCK2	Primary Prefrontal Cortical (PFC) neurons of neonatal rats	[36]
37.	Aβ-induced neurotoxicity	miR-34a-5p and miR-125b-5p	BACE1	Serum samples AD patients and Primary mouse cortical neurons (MCN) and Neuro2a (N2a) cells	[37]
38.	Propofol-induced Neurotoxicity	miR-215	LATS2	Neonatal rat hippocampal neuron	[38]
39.	Neurotoxicity induced by 6-hydroxydopamine	miR-107	PDCD10	In vitro and in vivo (Mouse, SH-SY5Y and PC12Â cells)	[39]
40.	Arsenic-induced Endoplasmic Reticulum Stress and Neurotoxicity	miR-124	SNPs - rs67543816 and rs35418153	Neural cells	[40]
41.	Amyloid-β induced neurotoxicity	miR-193a-3p	PTEN	Serum, cellular AD model of PC12 and SH-SY5Y	[41]

42	Aβ-induced neurotoxicity	miR-433	JAK2	Serum and cerebrospinal fluid (CS) of AD patients and AD cell model	[42]
43.	Propofol-induced neurotoxicity	miR-455-3p	EphA4	Primary hippocampal neurons of SD (Sprague-Dawley) rats	[43]
44.	Propofol-induced neurotoxicity	miR-582-5p	ROCK1	Primary rat hippocampal neurons	[44]
45.	Isoflurane-induced neurotoxicity	miR-24	p27kip1	Rat hippocampal neurons	[45]
46.	MPP (+)-induced neurotoxicity	miR-30b	SNCA	SH-SY5Y	[46]
47.	MPP (+)-induced neurotoxicity	miR-20a-5p	IRF9/NF-κB Axis	Hippocampal cell line-HT22 cells	[47]
48.	Isoflurane-induced neurotoxicity	miR-497	PLD1	Neonatal rat's hippocampus and hippocampal primary neuronal cell	[48]
49.	PD Neurotoxicity	miR-3473b	TREM2/ULK1	Mouse microglia cell line (BV2) cells	[49]
50.	Lidocaine induces neurotoxicity	miR-199a-5p	MYRF	Rat model	[50]
51.	Oxygen-glucose deprivation/reoxygenation (OGD/R) -induced neurotoxicity	miR-126	SIRT1/Nrf2 signaling pathway	Human umbilical vein endothelial cell (HUVEC)	[51]
52.	Sevoflurane-induced neurotoxicity	miR-1297	PTEN	Mice	[52]
53.	Bupivacaine-induced neurotoxicity	miR-494-3p	CDK6-PI3K/AKT Signaling	Primary mouse hippocampal neuronal cells (C57BL/6 mice)	[53]
54.	Ketamine-induced neurotoxicity	miR-429	BAG5	PC12 cells	[54]
55.	PD Neurotoxicity	miR-421	MEF2D	Cellular model of PD (Parkinson's disease)	[55]
56.	Amyloid-β induced neurotoxicity	miR-148a-3p	ROCK1	Serum of AD patients	[56]
57.	Isoflurane-induced neurotoxicity	miR-191	BDNF	In vitro and in vivo (hippocampal tissues of rats)	[57]
58.	Aβ1-42-induced neurotoxicity	miR-204-3p	Nox4	Hippocampus of APP/PS1 mice	[58]
59.	Aβ-induced neurotoxicity	miR-130a-3p	DAPK1	SH-SY5Y cells and hippocampus tissues of AD mice	[59]
60.	Isoflurane-induced neurotoxicity	miR-424-5p	FASN	hESC-derived neurons	[60]
61.	Sevoflurane-induced neurotoxicity	miR-221-3p	CDKN1B	Rat hippocampal neuron cells	[61]
62.	Sevoflurane-induced neurotoxicity	miR-128-3p	NOVA1	Rat hippocampal neuron cells	[62]
63.	Hypoxia/ischemia-induced	miR-381-3p	CCR2/NF-κB pathway	BV2 cells and HT-22 neurons	[63]

64.	Neurotoxicity induced by MPP+	miR-126-5p	SP1	SH-SY5Y and SK-N-SH cells	[64]
65.	Isoflurane-induced neurotoxicity	miR-128-3p	specificity protein 1 (SP1)	Sprague-Dawley (SD) rats	[65]
66.	Ischemic stroke (IS).	miR-107	FGF9/FGF12/ PI3K-AKT signaling pathway	Oxygen-Glucose Deprivation/Reoxygenation (OGD/R)-induced PC12 cells	[66]
67.	Sevoflurane-induced neurotoxicity	miR-384-3p	Aak1	Rat hippocampus	[67]
68.	Sevoflurane-induced neurotoxicity	miR-424	TLR4/MyD88/NF-κB pathway	Mouse and in PC12 cells	[68]
69.	Amyloid-β Induced Neurotoxicity	miR-26a-5p	PTGS2	Peripheral blood of AD patients and AD model cells (SH-SY5Y cells)	[69]
70.	Ketamine-induced neurotoxicity	miR-384-5p	GABRB1	Neonatal hippocampal neurons from rats	[70]
71.	Propofol-induced neurotoxicity	miR-17-5p	BCL2L11	SH-SY5Y cells	[71]
72.	Beta-Amyloid-Induced Neurotoxicity	miR-381-3p	PTGS2	Serum of AD patients and AD cell model- SH-SY5Y cells	[72]

Table S2. Potential modulator of miRNA as alleviative target for neurotoxicity.

Sr. No.	Modulators	MicroRNA	Neurotoxicity	References
1.	lncRNA-ATB	miR-200/ZNF217 axis	Amyloid-β-induced neurotoxicity	[73]
2.	Melatonin	miR-132/PTEN/AKT/FOXO3a pathway	Aβ-induced neurotoxicity	[74]
3.	LncRNA Rik-203	miR-101a-3p	Anesthesia neurotoxicity	[75]
4.	Baicalin	miR-192-5p	6-hydroxydopamine-induced neurotoxicity	[76]
5.	lncRNA MALAT1	miR-101-3p/PDCD4 axis	Bupivacaine-induced neurotoxicity	[77]
6.	Pramipexole	miR-494-3p/BDNF	MPP (+)-Induced Neurotoxicity	[78]
7.	LncRNA Rik-203	miR-466l-3p	Sevoflurane Induced Neurotoxicity	[79]
8.	Long-Noncoding RNA TUG1	MiR-152-3p/PTEN Pathway	Parkinson's Disease	[80]
9.	LncRNA H19	miR-585-3p/PIK3R3	MPTP-Induced Parkinson's Disease	[81]
10.	Vitexin	miR-409	Isoflurane-induced neurotoxicity	[82]
11.	Oxytocin	miR-26a/DAPK1 signaling pathway	MPTP-Induced Neurotoxicity	[83]
12.	Long Non-Coding RNA BACE1-AS	miR-214-3p	Isoflurane-Induced Neurotoxicity	[84]
13.	Dexmedetomidine	miR-381/LRRC4 /SDF-1/CXCR4 signaling pathway	Ropivacaine	[85]
14.	Long non-coding RNA Peg13	miR-128-3p	Sevoflurane toxicity	[86]

15.	lncRNA Gm15621	miR-133a/Sox4	Sevoflurane-induced neurotoxicity	[87]
16.	LncRNA KCNQ1OT1	miR-206	Ketamine-induced	[88]
17.	Berberine	circHDAC9/miR-142-5p axis	Aβ42-induced	[89]
18.	Long noncoding RNA small nucleolar RNA host gene 1 (SNHG1)	miR-181b	Sevoflurane-induced neurotoxicity	[90]
19.	Circular RNA circDLGAP4	miR-134-5p/CREB pathway	Parkinson's disease	[91]
20.	Neuron-derived exosomes	miR-124-3p	Neurotoxic microglia and astrocytes in Spinal cord injury (SCI)	[92]
21.	Syringin	miR-124-3p/BID Pathway	Aβ (25-35)-Induced Neurotoxicity	[93]
22.	lncRNA MAGI2-AS3	sponging miR-374b-5p	Amyloid-β induced neurotoxicity	[94]
23.	Dexmedetomidine	miR-330-3p/ULK1 axis	Sevoflurane-induced neurotoxicity	[95]
24.	LncRNA Neat1	miR-298-5p/Srpk1	Sevoflurane-Induced Neurotoxicity	[96]
25.	LINC00665	has-miR-34a-5p	Bupivacaine induced neurotoxicity	[97]
26.	lncRNA NEAT1	miR-374c-5p	MPTP-induced PD	[98]
27.	LncRNA Rian	miR-143-3p	Sevoflurane anesthesia-induced	[99]
28.	lipoxin A4 methyl ester (LXA4 ME)	miR-22/BAG5 pathway	Ketamine-induced neurotoxicity	[100]
29.	lncRNA ZFAS1	miR-421/zinc finger protein564 (ZNF564) axis	Bupivacaine-induced neurotoxicity	[101]
30.	LncRNA SNHG1	miR-181a-5p/CXCL12 axis	Neuronal injury in Parkinson's disease	[102]
31.	HOTAIR	miR-455-3p/NLRP1 axis	Propofol-induced neurotoxicity	[103]
32.	lncSh2d3c	mmu-miR-675-5p/Chmp4b/Bax axis	Mn-induced neurotoxicity	[104]
33.	Dexmedetomidine	miR-377-5p/Arc pathway	Propofol-induced	[105]
34.	Apple phenolic extracts	miR-22-3p/SIRT1 axis	Lead neurotoxicity	[106]
35.	Long Noncoding RNA H19	miR-17-5p	Isoflurane (ISO) neurotoxicity	[107]
36.	trametenolic acid B	mir-329-3p	Sevoflurane-induced	[108]
37.	Benzodiazepines	miR-133a-3p/EGFR pathway	Toxicity of lidocaine	[109]
38.	Long Noncoding RNA SNHG14	miR-519a-3p	MPP+ toxicity	[110]
39.	Bone Marrow Stem Cell-Exo-Derived TSG-6	STAT3/miR-7/NEDD4/LRRK2 Axis	1-Methyl-4-Phenylpyridinium+-Induced Neurotoxicity	[111]
40.	CircAXL	miR-1306-5p	Aβ (1-42)-Induced Neurotoxicity	[112]

41.	Myocardia-Related Transcription Factors-A (MRTF-A)	miR-1273g-3p/mTOR axis	Aβ-Induced Neurotoxicity	[113]
42.	LncRNA BDNF-AS	miR-9-5p/BACE1 pathway	neurotoxicity in Alzheimer's disease	[114]
43.	Circular RNA circ_0070441	miR-626/IRS2 axis.	MPP (+)-triggered neurotoxic effect	[115]
44.	LncRNA SNHG12	miR-497-5p	bupivacaine-induced neurotoxicity	[116]
45.	LncRNA Riken	MicroRNA-101a/MKP-1/JNK Pathway	sevoflurane-induced neurotoxic effects	[117]
46.	Resveratrol-Selenium Nanoparticles	Sirt1/miRNA-134/GSK3Î²	Neurotoxicity in a Rat Model of Alzheimer's Disease	[118]

Table S3. Potential miRNAs as alleviative target for AD related neurotoxicity.

Sr. No.	Neurotoxicity	miRNAs	Targets/Signalling Pathways	Experimental Validation Approach	References
1.	β -amyloid-induced neurotoxicity	miR-16	BACE1	AD tissues and the cellular AD model of PC12	[5]
2.	Amyloid-β-induced neurotoxicity	miR-107	BDNF-TrkB signaling pathway	mice	[11]
3.	Aβ-induced neurotoxicity	miR-34a	Caspase-2	SH-SY5Y cells	[17]
4.	Aβ-induced neurotoxicity	miR-409-5p	Plek	APP/PS1 mice cortexes	[18]
5.	Aβ-induced neurotoxicity	miR-29c-3p	TNFAIP1/ NF-κB signalling pathway	Neuroblastoma Cells	[28]
6.	Aβ (25-35)-induced neurotoxicity	miR-212	PDCD4/ PI3K/AKT signaling pathway	plasma from AD patients, SH-SY5Y and IMR-32 cells	[34]
7.	Aβ (25-35)-induced neurotoxicity	miR-33	Akt/mTOR pathway	SH-SY5Y cells	[35]
8.	Aβ-induced neurotoxicity	miR-34a-5p and miR-125b-5p	BACE1	Serum samples AD patients and Primary mouse cortical neurons (MCN) and Neuro2a (N2a) cells	[37]
9.	Amyloid-β induced neurotoxicity	miR-193a-3p	PTEN	Serum, cellular AD model of PC12 and SH-SY5Y	[41]
10.	Aβ-induced neurotoxicity	miR-433	JAK2	serum and cerebrospinal fluid (CS) of AD patients and AD cell model	[42]
11.	Amyloid-β induced neurotoxicity	miR-148a-3p	ROCK1	Serum of AD patients	[56]

12.	Aβ1-42-induced neurotoxicity	miR-204-3p	Nox4	hippocampus of APP/PS1 mice	[58]
13.	Aβ-induced neurotoxicity	miR-130a-3p	DAPK1	SH-SY5Y cells and hippocampus tissues of AD mice	[59]
14.	Amyloid-β Induced Neurotoxicity	miR-26a-5p	PTGS2	peripheral blood of AD patients and AD model cells (SH-SY5Y cells)	[69]
15.	Beta-Amyloid-Induced Neurotoxicity	miR-381-3p	PTGS2	Serum of AD patients and AD cell model- SH-SY5Y cells	[72]

Table S4. Potential miRNAs as alleviative target for PD related neurotoxicity.

Sr. No.	Neurotoxicity	miRNAs	Targets/ Signalling Pathways	Experimental Validation Approach	References
1.	MPP (+)-induced	miR-124-3p	STAT3	SH-SY5Y cells	[2]
2.	6-Hydroxydopamine-Induced Cell Model of PD	miR-124-3p	ANAX5	PC12 and SH-SY5Y cells	[7]
3.	Parkinson's Disease	miR-124	MEKK3	BV2 cells	[10]
4.	MPTP-induced Parkinson's disease	miR-190	Nlrp3	PD mouse model and BV2 cells	[15]
5.	Atrazine-induced Parkinson's disease	miR-7	BDNF/α-syn axis	peripheral blood and brain tissue in a rat model of PD	[19]
6.	(MPP+-) induced neurotoxicity	miR-9-5p	SIRT1	SH-SY5Ycells	[21]
7.	MPP+ neurotoxicity	miR-200a and miR-204	Sirt1 and BCL2	PC12 cells	[22]
8.	Parkinson's Disease	miR-326	KLK7-Mediated MAPK Signaling Pathway	mice with PD	[24]
9.	Parkinson's disease	miR-103a-3p	Parkin/Ambra1signaling	SH-SY5Y cells	[29]
10.	Neurotoxicity induced by 6-hydroxydopamine	miR-107	PDCD10	in vitro and in vivo (Mouse, SH-SY5Y and PC12Â cells)	[39]
11.	MPP (+)-induced neurotoxicity	miR-30b	SNCA	SH-SY5Y	[46]
12.	(MPP+-) induced neurotoxicity	miR-20a-5p	IRF9/NF-κB Axis	hippocampal cell line-HT22 cells	[47]
13.	PD	miR-3473b	TREM2/ULK1	moues microglia cell line (BV2) cells	[49]
14.	PD Neurotoxicity	miR-421	MEF2D	cellular model of PD (Parkinson's disease)	[55]
15.	Neurotoxicity induced by MPP+	miR-126-5p	SP1	SH-SY5Y and SK-N-SH cells	[64]

Table S5. Potential miRNAs as alleviative target for IS related neurotoxicity.

Sr. No.	Neurotoxicity	miRNAs	Targets/ Signalling Pathways	Experimental Validation Approach	References
1.	Hypoxia/ischemia-induced	miR-381-3p	CCR2/NF-κB pathway	BV2 cells and HT-22 neurons	[63]
2.	Ischemic stroke (IS)	miR-107	FGF9/FGF12/ PI3K-AKT signaling pathway	Oxygen-Glucose Deprivation/Reoxygenation (OGD/R)-induced PC12 cells	[66]

Table S6. Potential miRNAs as alleviative target for heavy metals related neurotoxicity.

Sr. No.	Neurotoxicity	miRNAs	Targets/ Signalling Pathways	Experimental Validation Approach	References

1.	Arsenic-Induced -neurotoxicity	miR-219	CaMKII	mouse hippocampus	[6]
2.	manganese induced neurotoxic	miR-7 and miR-433	SNCA and FGF-20 mRNA	SH-SY5Y cells	[12]
3.	Pb neurotoxicity	miR-106b-5p	XIAP	HT-22 and PC12 cells	[27]
4.	Arsenic-induced Endoplasmic Reticulum Stress and Neurotoxicity	miR-124	SNPs - rs67543816 and rs35418153	neural cells	[40]

Table S7. Potential miRNAs as alleviative target for other types of neurotoxicity.

Sr. No.	Neurotoxicity	miRNAs	Targets/ Signalling Pathways	Experimental Validation Approach	References
1.	Glutamate-induced neurotoxicity	miR-219	CaMKIIγ	rat hippocampal neurons	[4]
2.	Triazophos-induced toxicity	miR-217	nup43	adult zebrafish and ZF4 cells	[8]
3.	METH-mediated neurotoxicity	miR-142a-3p/miR-155-5p	Peli1	BV2 cells and brain of mouse	[20]
4.	T helper cell 1 (Th1)-skewed neurotoxicity	miR-494	HDAC2-STAT4 pathway	blood from AIS (acute ischemic stroke) patients and controls	[30]
5.	Lidocaine induces neurotoxicity	miR-199a-5p	MYRF	rat model	[50]
6.	Oxygen-glucose deprivation/reoxygenation (OGD/R) -induced neurotoxicity	miR-126	SIRT1/Nrf2 signaling pathway	human umbilical vein endothelial cell (HUVEC)	[51]

References

1. Lv, X.; Yan, J.; Jiang, J.; Zhou, X.; Lu, Y.; Jiang, H. MicroRNA-27a-3p Suppression of Peroxisome Proliferator-Activated Receptor-γ Contributes to Cognitive Impairments Resulting from Sevoflurane Treatment. *J Neurochem* **2017**, *143*, 306–319, doi:10.1111/jnc.14208.
2. Geng, L.; Liu, W.; Chen, Y. MiR-124-3p Attenuates MPP+-Induced Neuronal Injury by Targeting STAT3 in SH-SY5Y Cells. *Exp Biol Med (Maywood)* **2017**, *242*, 1757–1764, doi:10.1177/1535370217734492.
3. Chen, L.; Wang, X.; Huang, W.; Ying, T.; Chen, M.; Cao, J.; Wang, M. MicroRNA-137 and Its Downstream Target LSD1 Inversely Regulate Anesthetics-Induced Neurotoxicity in Dorsal Root Ganglion Neurons. *Brain Res Bull* **2017**, *135*, 1–7, doi:10.1016/j.brainresbull.2017.09.004.
4. Wang, T.; Cai, Q.; Yang, W.-J.; Fan, H.-H.; Yi, J.-F.; Xu, F. MicroRNA-219 Alleviates Glutamate-Induced Neurotoxicity in Cultured Hippocampal Neurons by Targeting Calmodulin-Dependent Protein Kinase II Gamma. *Neural Regen Res* **2018**, *13*, 1216–1224, doi:10.4103/1673-5374.235059.
5. Zhong, Z.; Yuan, K.; Tong, X.; Hu, J.; Song, Z.; Zhang, G.; Fang, X.; Zhang, W. MiR-16 Attenuates β-Amyloid-Induced Neurotoxicity through Targeting β-Site Amyloid Precursor Protein-Cleaving Enzyme 1 in an Alzheimer's Disease Cell Model. *Neuroreport* **2018**, *29*, 1365–1372, doi:10.1097/WNR.0000000000001118.
6. Wang, D.; Wang, X.; Liu, X.; Jiang, L.; Yang, G.; Shi, X.; Zhang, C.; Piao, F. Inhibition of MiR-219 Alleviates Arsenic-Induced Learning and Memory Impairments and Synaptic Damage Through Up-Regulating CaMKII in the Hippocampus. *Neurochem Res* **2018**, *43*, 948–958, doi:10.1007/s11064-018-2500-4.

7. Dong, R.-F.; Zhang, B.; Tai, L.-W.; Liu, H.-M.; Shi, F.-K.; Liu, N.-N. The Neuroprotective Role of MiR-124-3p in a 6-Hydroxydopamine-Induced Cell Model of Parkinson's Disease via the Regulation of ANAX5. *J Cell Biochem* **2018**, *119*, 269–277, doi:10.1002/jcb.26170.
8. Jia, L.; Zhang, D.; Huang, H.; Zhou, Y.; Zhou, S.; Guo, J. Triazophos-Induced Toxicity in Zebrafish: MiRNA-217 Inhibits Nup43. *Toxicol Res (Camb)* **2018**, *7*, 913–922, doi:10.1039/c8tx00065d.
9. Yang, L.; Shen, Q.; Xia, Y.; Lei, X.; Peng, J. Sevoflurane-induced Neurotoxicity Is Driven by OXR1 Post-transcriptional Downregulation Involving Hsa-miR-302e. *Mol Med Rep* **2018**, *18*, 4657–4665, doi:10.3892/mmr.2018.9442.
10. Yao, L.; Ye, Y.; Mao, H.; Lu, F.; He, X.; Lu, G.; Zhang, S. MicroRNA-124 Regulates the Expression of MEKK3 in the Inflammatory Pathogenesis of Parkinson's Disease. *J Neuroinflammation* **2018**, *15*, 13, doi:10.1186/s12974-018-1053-4.
11. Shu, B.; Zhang, X.; Du, G.; Fu, Q.; Huang, L. MicroRNA-107 Prevents Amyloid-β-Induced Neurotoxicity and Memory Impairment in Mice. *Int J Mol Med* **2018**, *41*, 1665–1672, doi:10.3892/ijmm.2017.3339.
12. Tarale, P.; Daiwile, A.P.; Sivanesan, S.; Stöger, R.; Bafana, A.; Naoghare, P.K.; Parmar, D.; Chakrabarti, T.; Krishnamurthi, K. Manganese Exposure: Linking down-Regulation of MiRNA-7 and MiRNA-433 with α-Synuclein Overexpression and Risk of Idiopathic Parkinson's Disease. *Toxicol In Vitro* **2018**, *46*, 94–101, doi:10.1016/j.tiv.2017.10.003.
13. Li, G.F.; Li, Z.B.; Zhuang, S.J.; Li, G.C. Inhibition of MicroRNA-34a Protects against Propofol Anesthesia-Induced Neurotoxicity and Cognitive Dysfunction via the MAPK/ERK Signaling Pathway. *Neurosci Lett* **2018**, *675*, 152–159, doi:10.1016/j.neulet.2018.03.052.
14. Jiang, J.D.; Zheng, X.C.; Huang, F.Y.; Gao, F.; You, M.Z.; Zheng, T. MicroRNA-107 Regulates Anesthesia-Induced Neural Injury in Embryonic Stem Cell Derived Neurons. *IUBMB Life* **2019**, *71*, 20–27, doi:10.1002/iub.1911.
15. Sun, Q.; Wang, S.; Chen, J.; Cai, H.; Huang, W.; Zhang, Y.; Wang, L.; Xing, Y. MicroRNA-190 Alleviates Neuronal Damage and Inhibits Neuroinflammation via Nlrp3 in MPTP-Induced Parkinson's Disease Mouse Model. *J Cell Physiol* **2019**, *234*, 23379–23387, doi:10.1002/jcp.28907.
16. Zhao, X.; Shu, F.; Wang, X.; Wang, F.; Wu, L.; Li, L.; Lv, H. Inhibition of MicroRNA-375 Ameliorated Ketamine-Induced Neurotoxicity in Human Embryonic Stem Cell Derived Neurons. *Eur J Pharmacol* **2019**, *844*, 56–64, doi:10.1016/j.ejphar.2018.11.035.
17. Li, Q.; Liu, T.; Yang, S.; Zhang, Z. Upregulation of MiR-34a by Inhibition of IRE1α Has Protective Effect against Aβ-Induced Injury in SH-SY5Y Cells by Targeting Caspase-2. *Oxid Med Cell Longev* **2019**, *2019*, 2140427, doi:10.1155/2019/2140427.
18. Guo, J.; Cai, Y.; Ye, X.; Ma, N.; Wang, Y.; Yu, B.; Wan, J. MiR-409-5p as a Regulator of Neurite Growth Is Down Regulated in APP/PS1 Murine Model of Alzheimer's Disease. *Front Neurosci* **2019**, *13*, 1264, doi:10.3389/fnins.2019.01264.
19. Li, B.; Jiang, Y.; Xu, Y.; Li, Y.; Li, B. Identification of MiRNA-7 as a Regulator of Brain-Derived Neurotrophic Factor/α-Synuclein Axis in Atrazine-Induced Parkinson's Disease by Peripheral Blood and Brain MicroRNA Profiling. *Chemosphere* **2019**, *233*, 542–548, doi:10.1016/j.chemosphere.2019.05.064.
20. Yu, G.; Song, Y.; Xie, C.; Tao, L.; Wan, F.; Jiang, L.; Wang, J.; Tang, J. MiR-142a-3p and MiR-155-5p Reduce Methamphetamine-Induced Inflammation: Role of the Target Protein Peli1. *Toxicol Appl Pharmacol* **2019**, *370*, 145–153, doi:10.1016/j.taap.2019.03.019.
21. Wang, Z.; Sun, L.; Jia, K.; Wang, H.; Wang, X. MiR-9-5p Modulates the Progression of Parkinson's Disease by Targeting SIRT1. *Neurosci Lett* **2019**, *701*, 226–233, doi:10.1016/j.neulet.2019.02.038.

22. Talepoor Ardakani, M.; Rostamian Delavar, M.; Baghi, M.; Nasr-Esfahani, M.H.; Kiani-Esfahani, A.; Ghaedi, K. Upregulation of MiR-200a and MiR-204 in MPP+ -Treated Differentiated PC12 Cells as a Model of Parkinson's Disease. *Mol Genet Genomic Med* **2019**, *7*, e548, doi:10.1002/mgg3.548.
23. Wu, Q.; Shang, Y.; Shen, T.; Liu, F.; Xu, Y.; Wang, H. Neuroprotection of MiR-214 against Isoflurane-Induced Neurotoxicity Involves the PTEN/PI3K/Akt Pathway in Human Neuroblastoma Cell Line SH-SY5Y. *Arch Biochem Biophys* **2019**, *678*, doi:10.1016/j.abb.2019.108181.
24. Zhang, Y.; Xu, W.; Nan, S.; Chang, M.; Fan, J. MicroRNA-326 Inhibits Apoptosis and Promotes Proliferation of Dopaminergic Neurons in Parkinson's Disease Through Suppression of KLK7-Mediated MAPK Signaling Pathway. *J Mol Neurosci* **2019**, *69*, 197–214, doi:10.1007/s12031-019-01349-1.
25. Shao, D.; Wu, Z.; Bai, S.; Fu, G.; Zou, Z. The Function of MiRNA-153 against Isoflurane-induced Neurotoxicity via Nrf2/ARE Cytoprotection. *Mol Med Rep* **2019**, *49*, 4001–4010, doi:10.3892/mmr.2019.10056.
26. Zhang, H.; Lin, J.; Hu, T.; Ren, Z.; Wang, W.; He, Q. Effect of MiR-132 on Bupivacaine-Induced Neurotoxicity in Human Neuroblastoma Cell Line. *J Pharmacol Sci* **2019**, *139*, 186–192, doi:10.1016/j.jphs.2019.01.014.
27. Xue, C.; Kang, B.; Su, P.; Wang, D.; Zhao, F.; Zhang, J.; Wang, X.; Lang, H.; Cao, Z. MicroRNA-106b-5p Participates in Lead (Pb2+)-Induced Cell Viability Inhibition by Targeting XIAP in HT-22 and PC12 Cells. *Toxicol In Vitro* **2020**, *66*, 104876, doi:10.1016/j.tiv.2020.104876.
28. Liu, Z.; Zhang, H.; Sun, L.; Zhu, K.; Lang, W. MiR-29c-3p Increases Cell Viability and Suppresses Apoptosis by Regulating the TNFAIP1/NF-KB Signaling Pathway via TNFAIP1 in Aβ-Treated Neuroblastoma Cells. *Neurochem Res* **2020**, *45*, 2375–2384, doi:10.1007/s11064-020-03096-x.
29. Zhou, J.; Zhao, Y.; Li, Z.; Zhu, M.; Wang, Z.; Li, Y.; Xu, T.; Feng, D.; Zhang, S.; Tang, F.; et al. MiR-103a-3p Regulates Mitophagy in Parkinson's Disease through Parkin/Ambra1 Signaling. *Pharmacol Res* **2020**, *160*, 105197, doi:10.1016/j.phrs.2020.105197.
30. Zhao, H.; Li, G.; Wang, R.; Tao, Z.; Ma, Q.; Zhang, S.; Han, Z.; Yan, F.; Li, F.; Liu, P.; et al. Silencing of MicroRNA-494 Inhibits the Neurotoxic Th1 Shift via Regulating HDAC2-STAT4 Cascade in Ischaemic Stroke. *Br J Pharmacol* **2020**, *177*, 128–144, doi:10.1111/bph.14852.
31. Liu, H.; Wang, J.; Yan, R.; Jin, S.; Wan, Z.; Cheng, J.; Li, N.; Chen, L.; Le, C. MicroRNA-204-5p Mediates Sevoflurane-Induced Cytotoxicity in HT22 Cells by Targeting Brain-Derived Neurotrophic Factor. *Histol Histopathol* **2020**, *35*, 1353–1361, doi:10.14670/HH-18-266.
32. Xu, L.; Xu, Q.; Xu, F.; Zhang, W.; Chen, Q.; Wu, H.; Chen, X. MicroRNA-325-3p Prevents Sevoflurane-Induced Learning and Memory Impairment by Inhibiting Nupr1 and C/EBPβ/IGFBP5 Signaling in Rats. *Aging* **2020**, *12*, 5209–5220, doi:10.18632/aging.102942.
33. Fan, D.; Yang, S.; Han, Y.; Zhang, R.; Yang, L. Isoflurane-Induced Expression of MiR-140-5p Aggravates Neurotoxicity in Diabetic Rats by Targeting SNX12. *J Toxicol Sci* **2020**, *45*, 69–76, doi:10.2131/jts.45.69.
34. Wang, Y.; Chang, Q. MicroRNA MiR-212 Regulates PDCD4 to Attenuate Aβ25-35-Induced Neurotoxicity via PI3K/AKT Signaling Pathway in Alzheimer's Disease. *Biotechnol Lett* **2020**, *42*, 1789–1797, doi:10.1007/s10529-020-02915-z.
35. Wang, X.; Li, X.; Huang, B.; Yang, L.; Chen, K.; Zhao, D.; Luo, X.; Wang, Y. Downregulation of MiR-33 Has Protective Effect Against $A\beta_{25-35}$-Induced Injury in SH-SH-SY5Y Cells. *Med Sci Monit* **2020**, *26*, e921026, doi:10.12659/MSM.921026.
36. Mao, Z.; Wang, W.; Gong, H.; Wu, Y.; Zhang, Y.; Wang, X. Upregulation of MiR-496 Rescues Propofol-Induced Neurotoxicity by Targeting Rho Associated Coiled-Coil Containing Protein Kinase 2 (ROCK2) in Prefrontal Cortical Neurons. *Curr Neurovasc Res* **2020**, *17*, 188–195, doi:10.2174/1567202617666200506101926.
37. Li, P.; Xu, Y.; Wang, B.; Huang, J.; Li, Q. MiR-34a-5p and MiR-125b-5p Attenuate Aβ-Induced Neurotoxicity through Targeting BACE1. *J Neurol Sci* **2020**, *413*, 116793, doi:10.1016/j.jns.2020.116793.

38. Tang, F.; Zhao, L.; Yu, Q.; Liu, T.; Gong, H.; Liu, Z.; Li, Q. Upregulation of MiR-215 Attenuates Propofol-Induced Apoptosis and Oxidative Stress in Developing Neurons by Targeting LATS2. *Molecular Medicine* **2020**, *26*, doi:10.1186/s10020-020-00170-6.
39. Sun, L.; Zhang, T.; Xiu, W.; Cao, W.; He, M.; Sun, W.; Zhao, W. MiR-107 Overexpression Attenuates Neurotoxicity Induced by 6-Hydroxydopamine Both in Vitro and in Vivo. *Chem Biol Interact* **2020**, *315*, 108908, doi:10.1016/j.cbi.2019.108908.
40. Park, H.R.; Sun, R.; Panganiban, R.A.; Christiani, D.C.; Lu, Q. MicroRNA-124 Reduces Arsenic-Induced Endoplasmic Reticulum Stress and Neurotoxicity and Is Linked with Neurodevelopment in Children. *Sci Rep* **2020**, *10*, doi:10.1038/s41598-020-62594-8.
41. Cao, F.; Liu, Z.; Sun, G. Diagnostic Value of MiR-193a-3p in Alzheimer's Disease and MiR-193a-3p Attenuates Amyloid-β Induced Neurotoxicity by Targeting PTEN. *Exp Gerontol* **2020**, *130*, 110814, doi:10.1016/j.exger.2019.110814.
42. Wang, R.; Zhang, J. Clinical Significance of MiR-433 in the Diagnosis of Alzheimer's Disease and Its Effect on Aβ-Induced Neurotoxicity by Regulating JAK2. *Exp Gerontol* **2020**, *141*, 111080, doi:10.1016/j.exger.2020.111080.
43. Zhu, X.; Li, H.; Tian, M.; Zhou, S.; He, Y.; Zhou, M. MiR-455-3p Alleviates Propofol-Induced Neurotoxicity by Reducing EphA4 Expression in Developing Neurons. *Biomarkers* **2020**, *25*, 685–692, doi:10.1080/1354750X.2020.1832147.
44. Zhang, Z.; Xu, Y.; Chi, S.; Cui, L. MicroRNA-582-5p Reduces Propofol-Induced Apoptosis in Developing Neurons by Targeting ROCK1. *Curr Neurovasc Res* **2020**, *17*, 140–146, doi:10.2174/1567202617666200207124817.
45. Li, N.; Yue, L.; Wang, J.; Wan, Z.; Bu, W. MicroRNA-24 Alleviates Isoflurane-Induced Neurotoxicity in Rat Hippocampus via Attenuation of Oxidative Stress. *Biochem Cell Biol* **2020**, *98*, 208–218, doi:10.1139/bcb-2019-0188.
46. Shen, Y.-F.; Zhu, Z.-Y.; Qian, S.-X.; Xu, C.-Y.; Wang, Y.-P. MiR-30b Protects Nigrostriatal Dopaminergic Neurons from MPP(+)-Induced Neurotoxicity via SNCA. *Brain Behav* **2020**, *10*, e01567, doi:10.1002/brb3.1567.
47. Wang, Q.; Wang, Y.; Zhou, F.; Li, J.; Lu, G.; Zhao, Y. MiR-20a-5p Regulates MPP+-Induced Oxidative Stress and Neuroinflammation in HT22 Cells by Targeting IRF9/NF-KB Axis. *Evid Based Complement Alternat Med* **2021**, *2021*, 6621206, doi:10.1155/2021/6621206.
48. Que, Y.; Zhang, F.; Peng, J.; Zhang, Z.; Zhang, D.; He, M. Repeated Isoflurane Exposures of Neonatal Rats Contribute to Cognitive Dysfunction in Juvenile Animals: The Role of Mir-497 in Isoflurane-Induced Neurotoxicity. *Folia Histochem Cytobiol* **2021**, *59*, 114–123, doi:10.5603/FHC.a2021.0011.
49. Lv, Q.; Zhong, Z.; Hu, B.; Yan, S.; Yan, Y.; Zhang, J.; Shi, T.; Jiang, L.; Li, W.; Huang, W. MicroRNA-3473b Regulates the Expression of TREM2/ULK1 and Inhibits Autophagy in Inflammatory Pathogenesis of Parkinson Disease. *J Neurochem* **2021**, *157*, 599–610, doi:10.1111/jnc.15299.
50. Zhang, Z.; Wang, J.; Song, Z.; Wang, Y.; Cheng, Z.; Guo, Q.; Wang, E.; Jian, Y.; Wu, L. Downregulation of MicroRNA-199a-5p Alleviated Lidocaine-Induced Sensory Dysfunction and Spinal Cord Myelin Lesions in a Rat Model. *Toxicol Lett* **2021**, *336*, 1–10, doi:10.1016/j.toxlet.2020.11.001.
51. Li, J.; Yang, C.; Wang, Y. MiR-126 Overexpression Attenuates Oxygen-glucose Deprivation/Reperfusion Injury by Inhibiting Oxidative Stress and Inflammatory Response via the Activation of SIRT1/Nrf2 Signaling Pathway in Human Umbilical Vein Endothelial Cells. *Mol Med Rep* **2021**, *23*, doi:10.3892/mmr.2020.11804.
52. Wang, Q.; Luo, J.; Sun, R.; Liu, J. MicroRNA-1297 Suppressed the Akt/GSK3β Signaling Pathway and Stimulated Neural Apoptosis in an in Vivo Sevoflurane Exposure Model. *Journal of International Medical Research* **2021**, *49*, doi:10.1177/0300060520982104.
53. Zhang, L.; Zhang, L.; Guo, F. MiRNA-494-3p Regulates Bupivacaine-Induced Neurotoxicity by the CDK6-PI3K/AKT Signaling. *Neurotox Res* **2021**, *39*, 2007–2017, doi:10.1007/s12640-021-00427-w.

54. Fan, X.; Bian, W.; Liu, M.; Li, J.; Wang, Y. MiRNA-429 Alleviates Ketamine-Induced Neurotoxicity through Targeting BAG5. *Environ Toxicol* **2021**, *36*, 620–627, doi:10.1002/tox.23066.
55. Dong, Y.; Xiong, J.; Ji, L.; Xue, X. MiR-421 Aggravates Neurotoxicity and Promotes Cell Death in Parkinson's Disease Models by Directly Targeting MEF2D. *Neurochem Res* **2021**, *46*, 299–308, doi:10.1007/s11064-020-03166-0.
56. Zhang, H.; Liu, W.; Ge, H.; Li, K. Aberrant Expression of MiR-148a-3p in Alzheimer's Disease and Its Protective Role against Amyloid-β Induced Neurotoxicity. *Neurosci Lett* **2021**, *756*, 135953, doi:10.1016/j.neulet.2021.135953.
57. Li, H.; Du, M.; Xu, W.; Wang, Z. MiR-191 Downregulation Protects against Isoflurane-Induced Neurotoxicity through Targeting BDNF. *Toxicol Mech Methods* **2021**, *31*, 367–373, doi:10.1080/15376516.2021.1886211.
58. Tao, W.; Yu, L.; Shu, S.; Liu, Y.; Zhuang, Z.; Xu, S.; Bao, X.; Gu, Y.; Cai, F.; Song, W.; et al. MiR-204-3p/Nox4 Mediates Memory Deficits in a Mouse Model of Alzheimer's Disease. *Mol Ther* **2021**, *29*, 396–408, doi:10.1016/j.ymthe.2020.09.006.
59. Wang, Y.; Shi, M.; Hong, Z.; Kang, J.; Pan, H.; Yan, C. MiR-130a-3p Has Protective Effects in Alzheimer's Disease via Targeting DAPK1. *Am J Alzheimers Dis Other Demen 36*, 15333175211020572, doi:10.1177/15333175211020572.
60. Gu, X.; Yue, W.; Xiu, M.; Zhang, Q.; Xie, R. MicroRNA-424-5p Alleviates Isoflurane Anesthesia-Induced Neurotoxicity in Human Embryonic Stem Cell-Derived Neurons by Targeting FASN. *Comput Math Methods Med* **2022**, *2022*, doi:10.1155/2022/2517463.
61. Wang, Q.; Tian, X.; Lu, Q.; Liu, K.; Gong, J. Study on the Ameliorating Effect of MiR-221-3p on the Nerve Cells Injury Induced by Sevoflurane. *Int J Neurosci* **2022**, *132*, 181–191, doi:10.1080/00207454.2020.1806267.
62. Li, D.; Sun, J.; Yu, M.; Wang, Y.; Lu, Y.; Li, B. The Protective Effects of MiR-128-3p on Sevoflurane-Induced Progressive Neurotoxicity in Rats by Targeting NOVA1. *J Toxicol Sci* **2022**, *47*, 51–60, doi:10.2131/jts.47.51.
63. Che, Y.; He, J.; Li, X.; Wu, D.; Zhang, Y.; Yuan, G. Overexpression of MicroRNA-381-3p Ameliorates Hypoxia/Ischemia-Induced Neuronal Damage and Microglial Inflammation via Regulating the C-C Chemokine Receptor Type 2 /Nuclear Transcription Factor-Kappa B Axis. *Bioengineered* **2022**, *13*, 6839–6855, doi:10.1080/21655979.2022.2038448.
64. Han, Y.-P.; Liu, Z.-J.; Bao, H.-H.; Wang, Q.; Su, L.-L. MiR-126-5p Targets SP1 to Inhibit the Progression of Parkinson's Disease. *Eur Neurol* **2022**, *85*, 235–244, doi:10.1159/000521525.
65. Qian, D.; Dai, S.; Sun, Y.; Yuan, Y.; Wang, L. MiR-128-3p Attenuates the Neurotoxicity in Rats Induced by Isoflurane Anesthesia. *Neurotox Res* **2022**, *40*, 714–720, doi:10.1007/s12640-022-00512-8.
66. Fan, Q.; Wu, M.; Li, C.; Li, J. MiR-107 Aggravates Oxygen-Glucose Deprivation/Reoxygenation (OGD/R)-Induced Injury Through Inactivating PI3K-AKT Signalling Pathway by Targeting FGF9/FGF12 in PC12 Cells. *J Stroke Cerebrovasc Dis* **2022**, *31*, 106295, doi:10.1016/j.jstrokecerebrovasdis.2021.106295.
67. Chen, Y.; Gao, X.; Pei, H. MiRNA-384-3p Alleviates Sevoflurane-Induced Nerve Injury by Inhibiting Aak1 Kinase in Neonatal Rats. *Brain Behav* **2022**, *12*, doi:10.1002/brb3.2556.
68. Li, Z.; Wang, T.; Yu, Y. MiR-424 Inhibits Apoptosis and Inflammatory Responses Induced by Sevoflurane through TLR4/MyD88/NF-KB Pathway. *BMC Anesthesiol* **2022**, *22*, doi:10.1186/s12871-022-01590-z.
69. Xie, T.; Pei, Y.; Shan, P.; Xiao, Q.; Zhou, F.; Huang, L.; Wang, S. Identification of MiRNA-MRNA Pairs in the Alzheimer's Disease Expression Profile and Explore the Effect of MiR-26a-5p/PTGS2 on Amyloid-β Induced Neurotoxicity in Alzheimer's Disease Cell Model. *Front Aging Neurosci* **2022**, *14*, 909222, doi:10.3389/fnagi.2022.909222.
70. Yang, Q.; Long, F. MiRNA-384-5p Targets GABRB1 to Regulate Ketamine-Induced Neurotoxicity in Neurons. *Turk Neurosurg* **2022**, doi:10.5137/1019-5149.JTN.36367-21.2.
71. Xiu, M.; Luan, H.; Gu, X.; Liu, C.; Xu, D. MicroRNA-17-5p Protects against Propofol Anesthesia-Induced Neurotoxicity and Autophagy Impairment via Targeting BCL2L11. *Comput Math Methods Med* **2022**, *2022*, doi:10.1155/2022/6018037.

72. Zhang, M.; Liu, Y.; Teng, P.; Yang, Q. Differential Expression of MiR-381-3p in Alzheimer's Disease Patients and Its Role in Beta-Amyloid-Induced Neurotoxicity and Inflammation. *Neuroimmunomodulation* **2022**, *29*, 211–219, doi:10.1159/000519780.
73. Wang, J.; Zhou, T.; Wang, T.; Wang, B. Suppression of LncRNA-ATB Prevents Amyloid-β-Induced Neurotoxicity in PC12 Cells via Regulating MiR-200/ZNF217 Axis. *Biomedicine & Pharmacotherapy* **2018**, *108*, 707–715, doi:10.1016/j.biopha.2018.08.155.
74. Zhao, Y.; Zhao, R.; Wu, J.; Wang, Q.; Pang, K.; Shi, Q.; Gao, Q.; Hu, Y.; Dong, X.; Zhang, J.; et al. Melatonin Protects against Aβ-Induced Neurotoxicity in Primary Neurons via MiR-132/PTEN/AKT/FOXO3a Pathway. *Biofactors* **2018**, *44*, 609–618, doi:10.1002/biof.1411.
75. Zhang, L.; Yan, J.; Liu, Q.; Xie, Z.; Jiang, H. LncRNA Rik-203 Contributes to Anesthesia Neurotoxicity via MicroRNA-101a-3p and GSK-3β-Mediated Neural Differentiation. *Sci Rep* **2019**, *9*, 6822, doi:10.1038/s41598-019-42991-4.
76. Kang, C.; Wang, L.; Kang, M.; Liu, X.; Fu, Y.; Gao, J. Baicalin Alleviates 6-Hydroxydopamine-Induced Neurotoxicity in PC12 Cells by down-Regulation of MicroRNA-192-5p. *Brain Res* **2019**, *1708*, 84–92, doi:10.1016/j.brainres.2018.12.015.
77. Zhao, Y.; Ai, Y. Knockdown of LncRNA MALAT1 Alleviates Bupivacaine-Induced Neurotoxicity via the MiR-101-3p/PDCD4 Axis. *Life Sci* **2019**, *232*, 116606, doi:10.1016/j.lfs.2019.116606.
78. Deng, C.; Zhu, J.; Yuan, J.; Xiang, Y.; Dai, L. Pramipexole Inhibits MPP+-Induced Neurotoxicity by MiR-494-3p/BDNF. *Neurochem Res* **2020**, *45*, 268–277, doi:10.1007/s11064-019-02910-5.
79. Zhang, L.; Xue, Z.; Yan, J.; Jiang, H. LncRNA Rik-203 Contributes to Sevoflurane Induced Neurotoxicity? *Front Med (Lausanne)* **2020**, *7*, 353, doi:10.3389/fmed.2020.00353.
80. Zhai, K.; Liu, B.; Gao, L. Long-Noncoding RNA TUG1 Promotes Parkinson's Disease via Modulating MiR-152-3p/PTEN Pathway. *Hum Gene Ther* **2020**, *31*, 1274–1287, doi:10.1089/hum.2020.106.
81. Zhang, Y.; Xia, Q.; Lin, J. LncRNA H19 Attenuates Apoptosis in MPTP-Induced Parkinson's Disease Through Regulating MiR-585-3p/PIK3R3. *Neurochem Res* **2020**, *45*, 1700–1710, doi:10.1007/s11064-020-03035-w.
82. Qi, Y.; Chen, L.; Shan, S.; Nie, Y.; Wang, Y. Vitexin Improves Neuron Apoptosis and Memory Impairment Induced by Isoflurane via Regulation of MiR-409 Expression. *Adv Clin Exp Med* **2020**, *29*, 135–145, doi:10.17219/acem/104556.
83. Almansoub, H.A.M.M.; Tang, H.; Wu, Y.; Wang, D.-Q.; Mahaman, Y.A.R.; Salissou, M.T.M.; Lu, Y.; Hu, F.; Zhou, L.-T.; Almansob, Y.A.M.; et al. Oxytocin Alleviates MPTP-Induced Neurotoxicity in Mice by Targeting MicroRNA-26a/Death-Associated Protein Kinase 1 Pathway. *J Alzheimers Dis* **2020**, *74*, 883–901, doi:10.3233/JAD-191091.
84. He, W.; Chi, S.; Jin, X.; Lu, J.; Zheng, W.; Yan, J.; Zhang, D. Long Non-Coding RNA BACE1-AS Modulates Isoflurane-Induced Neurotoxicity to Alzheimer's Disease Through Sponging MiR-214-3p. *Neurochem Res* **2020**, *45*, 2324–2335, doi:10.1007/s11064-020-03091-2.
85. Xue, Y.; Xu, T.; Jiang, W. Dexmedetomidine Protects PC12 Cells from Ropivacaine Injury through MiR-381/LRRC4 /SDF-1/CXCR4 Signaling Pathway. *Regen Ther* **2020**, *14*, 322–329, doi:10.1016/j.reth.2020.03.001.
86. Jiang, Y.; Wang, Y.; Sun, Y.; Jiang, H. Long Non-Coding RNA Peg13 Attenuates the Sevoflurane Toxicity against Neural Stem Cells by Sponging MicroRNA-128-3p to Preserve Sox13 Expression. *PLoS One* **2020**, *15*, e0243644, doi:10.1371/journal.pone.0243644.
87. Zhao, Y.; Ai, Y. Overexpression of LncRNA Gm15621 Alleviates Apoptosis and Inflammation Response Resulting from Sevoflurane Treatment through Inhibiting MiR-133a/Sox4. *J Cell Physiol* **2020**, *235*, 957–965, doi:10.1002/jcp.29011.

88. Yao, Y.; Wang, X.; Gao, J. LncRNA KCNQ1OT1 Sponges MiR-206 to Ameliorate Neural Injury Induced by Anesthesia via Up-Regulating BDNF. *Drug Des Devel Ther* **2020**, *14*, 4789–4800, doi:10.2147/DDDT.S256319.
89. Zhang, N.; Gao, Y.; Yu, S.; Sun, X.; Shen, K. Berberine Attenuates Aβ42-Induced Neuronal Damage through Regulating CircHDAC9/MiR-142-5p Axis in Human Neuronal Cells. *Life Sci* **2020**, *252*, 117637, doi:10.1016/j.lfs.2020.117637.
90. Zhang, N.; Wang, D.; Yang, X.; Hou, D. Long Noncoding RNA Small Nucleolar RNA Host Gene 1 Contributes to Sevoflurane-Induced Neurotoxicity through Negatively Modulating MicroRNA-181b. *Neuroreport* **2020**, *31*, 416–424, doi:10.1097/WNR.0000000000001430.
91. Feng, Z.; Zhang, L.; Wang, S.; Hong, Q. Circular RNA CircDLGAP4 Exerts Neuroprotective Effects via Modulating MiR-134-5p/CREB Pathway in Parkinson's Disease. *Biochem Biophys Res Commun* **2020**, *522*, 388–394, doi:10.1016/j.bbrc.2019.11.102.
92. Jiang, D.; Gong, F.; Ge, X.; Lv, C.; Huang, C.; Feng, S.; Zhou, Z.; Rong, Y.; Wang, J.; Ji, C.; et al. Neuron-Derived Exosomes-Transmitted MiR-124-3p Protect Traumatically Injured Spinal Cord by Suppressing the Activation of Neurotoxic Microglia and Astrocytes. *J Nanobiotechnology* **2020**, *18*, 105, doi:10.1186/s12951-020-00665-8.
93. Zhang, N.; Zhao, L.; Su, Y.; Liu, X.; Zhang, F.; Gao, Y. Syringin Prevents Aβ25-35-Induced Neurotoxicity in SK-N-SH and SK-N-BE Cells by Modulating MiR-124-3p/BID Pathway. *Neurochem Res* **2021**, *46*, 675–685, doi:10.1007/s11064-021-03240-1.
94. Zhang, J.; Wang, R. Deregulated LncRNA MAGI2-AS3 in Alzheimer's Disease Attenuates Amyloid-β Induced Neurotoxicity and Neuroinflammation by Sponging MiR-374b-5p. *Exp Gerontol* **2021**, *144*, 111180, doi:10.1016/j.exger.2020.111180.
95. Xu, S.; Gao, R.; Chen, L. Dexmedetomidine Regulates Sevoflurane-Induced Neurotoxicity through the MiR-330-3p/ULK1 Axis. *J Biochem Mol Toxicol* **2021**, *35*, e22919, doi:10.1002/jbt.22919.
96. Wei, X.; Xu, S.; Chen, L. LncRNA Neat1/MiR-298-5p/Srpk1 Contributes to Sevoflurane-Induced Neurotoxicity. *Neurochem Res* **2021**, *46*, 3356–3364, doi:10.1007/s11064-021-03436-5.
97. Yang, Z.; Hu, S.; He, Y.; Ji, L. LINC00665 Rescues Bupivacaine Induced Neurotoxicity in Human Neural Cell of SH-SY5Y through Has-MiR-34a-5p. *Brain Res Bull* **2021**, *177*, 210–216, doi:10.1016/j.brainresbull.2021.10.004.
98. Dong, L.I.; Zheng, Y.; Gao, L.; Luo, X. LncRNA NEAT1 Prompts Autophagy and Apoptosis in MPTP-Induced Parkinson's Disease by Impairing MiR-374c-5p. *Acta Biochim Biophys Sin (Shanghai)* **2021**, *53*, 870–882, doi:10.1093/abbs/gmab055.
99. Yu, Y.; Zhang, W.; Zhu, D.; Wang, H.; Shao, H.; Zhang, Y. LncRNA Rian Ameliorates Sevoflurane Anesthesia-Induced Cognitive Dysfunction through Regulation of MiR-143-3p/LIMK1 Axis. *Hum Cell* **2021**, *34*, 808–818, doi:10.1007/s13577-021-00502-6.
100. Wang, X.-S.; Li, L.-C.; Zhang, X.; Gao, J. Lipoxin A4 Methyl Ester Protects PC12 Cells from Ketamine-Induced Neurotoxicity via the MiR-22/BAG5 Pathway. *Hum Exp Toxicol* **2021**, *40*, S519–S529, doi:10.1177/09603271211051602.
101. Yuan, L.; Xu, H.; Guo, R.; Lu, T.; Li, X. Long Non-Coding RNA ZFAS1 Alleviates Bupivacaine-Induced Neurotoxicity by Regulating the MiR-421/Zinc Finger Protein564 (ZNF564) Axis. *Bioengineered* **2021**, *12*, 5231–5240, doi:10.1080/21655979.2021.1960776.
102. Wang, H.; Wang, X.; Zhang, Y.; Zhao, J. LncRNA SNHG1 Promotes Neuronal Injury in Parkinson's Disease Cell Model by MiR-181a-5p/CXCL12 Axis. *J Mol Histol* **2021**, *52*, 153–163, doi:10.1007/s10735-020-09931-3.
103. Gong, H.; Wan, X.; Zhang, Y.; Liang, S. Downregulation of HOTAIR Reduces Neuronal Pyroptosis by Targeting MiR-455-3p/NLRP1 Axis in Propofol-Treated Neurons in Vitro. *Neurochem Res* **2021**, *46*, 1141–1150, doi:10.1007/s11064-021-03249-6.

104. Lu, W.; Li, D.; Wei, S.; Yang, J.; Tang, C.; Ma, X.; Zou, Y. Long Noncoding RNA Sh2d3c Promotes Manganese-Induced Neuronal Apoptosis through the Mmu-MiR-675-5p/Chmp4b/Bax Axis. *Toxicol Lett* **2022**, *365*, 24–35, doi:10.1016/j.toxlet.2022.06.002.
105. Chen, Z.; Ding, Y.; Zeng, Y.; Zhang, X.-P.; Chen, J.-Y. Dexmedetomidine Reduces Propofol-Induced Hippocampal Neuron Injury by Modulating the MiR-377-5p/Arc Pathway. *BMC Pharmacol Toxicol* **2022**, *23*, 18, doi:10.1186/s40360-022-00555-9.
106. Ren, Y.; Sun-Waterhouse, D.; Ouyang, F.; Tan, X.; Li, D.; Xu, L.; Li, B.; Wang, Y.; Li, F. Apple Phenolic Extracts Ameliorate Lead-Induced Cognitive Impairment and Depression- and Anxiety-like Behavior in Mice by Abating Oxidative Stress, Inflammation and Apoptosis via the MiR-22-3p/SIRT1 Axis. *Food Funct* **2022**, *13*, 2647–2661, doi:10.1039/d1fo03750a.
107. Ge, Y.; Lin, D.; Cui, B.; Zhang, L.; Li, S.; Wang, Z.; Ma, J. Effects of Long Noncoding RNA H19 on Isoflurane-Induced Cognitive Dysregulation by Promoting Neuroinflammation. *Neuroimmunomodulation* **2022**, *29*, 117–127, doi:10.1159/000519124.
108. Chen, J.; Feng, S.; Li, L.; Qiu, S.; Jin, Y.; Sun, Y. Protective Role of Trametenolic Acid B against Sevoflurane-Induced Cognitive Impairments by Its Different Regulatory Modalities of Mir-329-3p in Neurons and Microglia. *Mol Med* **2022**, *28*, 77, doi:10.1186/s10020-022-00477-6.
109. Li, Y.; Yi, L.; Li, X.; Li, Y. Benzodiazepines Safeguards Nerve Cells from the Toxicity of Lidocaine via MiR-133a-3p/EGFR Pathway. *Transpl Immunol* **2022**, *71*, 101510, doi:10.1016/j.trim.2021.101510.
110. Zhuang, Z.; Zhang, L.; Liu, C. SNHG14 Upregulation Was a Molecular Mechanism Underlying MPP+ Neurotoxicity in Dopaminergic SK-N-SH Cells via SNHG14-MiR-519a-3p-ATG10 CeRNA Pathway. *Neurotox Res* **2022**, *40*, 553–563, doi:10.1007/s12640-022-00488-5.
111. Huang, D.; Zhang, M.; Tan, Z. Bone Marrow Stem Cell-Exo-Derived TSG-6 Attenuates 1-Methyl-4-Phenylpyridinium+-Induced Neurotoxicity via the STAT3/MiR-7/NEDD4/LRRK2 Axis. *J Neuropathol Exp Neurol* **2022**, *81*, 621–634, doi:10.1093/jnen/nlac049.
112. Meng, S.; Wang, B.; Li, W. CircAXL Knockdown Alleviates Aβ1-42-Induced Neurotoxicity in Alzheimer's Disease via Repressing PDE4A by Releasing MiR-1306-5p. *Neurochem Res* **2022**, *47*, 1707–1720, doi:10.1007/s11064-022-03563-7.
113. Zhang, W.; Yang, Y.; Xiang, Z.; Cheng, J.; Yu, Z.; Wang, W.; Hu, L.; Ma, F.; Deng, Y.; Jin, Z.; et al. MRTF-A-Mediated Protection against Amyloid-β-Induced Neuronal Injury Correlates with Restoring Autophagy via MiR-1273g-3p/MTOR Axis in Alzheimer Models. *Aging* **2022**, *14*, 4305–4325, doi:10.18632/aging.203883.
114. Ding, Y.; Luan, W.; Shen, X.; Wang, Z.; Cao, Y. LncRNA BDNF-AS as CeRNA Regulates the MiR-9-5p/BACE1 Pathway Affecting Neurotoxicity in Alzheimer's Disease. *Arch Gerontol Geriatr* **2022**, *99*, 104614, doi:10.1016/j.archger.2021.104614.
115. Cao, X.; Guo, J.; Mochizuki, H.; Xu, D.; Zhang, T.; Han, H.; Ma, T.; Qi, M.; He, J. Circular RNA Circ_0070441 Regulates MPP+-Triggered Neurotoxic Effect in SH-SY5Y Cells via MiR-626/IRS2 Axis. *Metab Brain Dis* **2022**, *37*, 513–524, doi:10.1007/s11011-021-00869-3.
116. Sun, L.; Yuan, R. LncRNA SNHG12 Ameliorates Bupivacaine-Induced Neurotoxicity by Sponging MiR-497-5p to Upregulate NLRX1. *Hum Exp Toxicol* **2022**, *41*, 9603271221089000, doi:10.1177/09603271221089001.
117. Hou, Q.; Li, S.; Zhang, B.; Chu, H.; Ni, C.; Fei, X.; Zheng, H. LncRNA Riken Attenuated Sevoflurane-Induced Neuroinflammation by Regulating the MicroRNA-101a/MKP-1/JNK Pathway. *Neurotox Res* **2022**, *40*, 186–197, doi:10.1007/s12640-021-00443-w.
118. Abozaid, O.A.R.; Sallam, M.W.; El-Sonbaty, S.; Aziza, S.; Emad, B.; Ahmed, E.S.A. Resveratrol-Selenium Nanoparticles Alleviate Neuroinflammation and Neurotoxicity in a Rat Model of Alzheimer's Disease by Regulating Sirt1/MiRNA-134/GSK3β Expression. *Biol Trace Elem Res* **2022**, doi:10.1007/s12011-021-03073-7.
